# Taphonomy of *Isisfordia duncani* specimens from the Lower Cretaceous (upper Albian) portion of the Winton Formation, Isisford, central-west Queensland

**DOI:** 10.1098/rsos.171651

**Published:** 2018-03-28

**Authors:** Caitlin E. Syme, Steven W. Salisbury

**Affiliations:** School of Biological Sciences, The University of Queensland, Brisbane, Queensland, Australia

**Keywords:** *Isisfordia duncani*, crocodyliforms, taphonomy, concretion, Winton Formation, decay

## Abstract

Taphonomic analysis of fossil material can benefit from including the results of actualistic decay experiments. This is crucial in determining the autochthony or allochthony of fossils of juvenile and adult *Isisfordia duncani*, a basal eusuchian from the Lower Cretaceous (upper Albian) distal-fluvial-deltaic lower Winton Formation near Isisford. The taphonomic characteristics of the *I. duncani* fossils were documented using a combination of traditional taphonomic analysis alongside already published actualistic decay data from juvenile *Crocodylus porosus* carcasses. We found that the *I. duncani* holotype, paratypes and referred specimens show little signs of weathering and no signs of abrasion. Disarticulated skeletal elements are often found in close proximity to the rest of the otherwise articulated skeleton. The isolated and disarticulated skeletal elements identified, commonly cranial, maxillary and mandibular elements, are typical of lag deposits. The holotype QM F36211 and paratype QM F34642 were classified as autochthonous, and the remaining *I. duncani* paratypes and referred specimens are parautochthonous. We propose that *I. duncani* inhabited upper and lower delta plains near the Eromanga Sea in life. Their carcasses were buried in sediment-laden floodwaters in delta plain overbank and distributary channel deposits. Future studies should refer to *I. duncani* as a brackish water tolerant species.

## 1. Introduction

Fossil remains of crocodyliforms, non-avian dinosaurs and osteichthyans encased in calcite-cemented sandstone concretions have been recovered from the Lower Cretaceous (upper Albian) portion of the Winton Formation near Isisford in central-west Queensland since the mid-1990s. Taxa formally described from this site include the ichthyodectiform fish *Cladocyclus geddesi* [1] and the basal eusuchian crocodyliform *Isisfordia duncani* [2] (see Figueiredo *et al*. [3], Turner & Pritchard [4] and Turner [5] for alternative phylogenetic placements of *I. duncani*). *Isisfordia duncani* is represented by multiple semi-articulated and partially complete skeletons, isolated partially articulated forelimbs and hindlimbs with associated osteoderms, an isolated skull, plus isolated and associated cranial and postcranial elements. Detailed outcrop and core-based sedimentological and stratigraphic investigations of the Winton Formation at Isisford indicate that these remains were deposited in a fluvial to tidally influenced upper and/or lower delta plain proximal to the Eromanga Sea [6,7]. However, whether these crocodyliform fossils represent animals that died and were buried in the same environment (autochthonous) or instead are carcasses that were transported from another environment (parautochthonous or allochthonous) (*sensu* Kidwell *et al*. [8]) has not yet been ascertained. Fossil provenance and inferred habitat preference in life is crucial for palaeoenvironmental reconstructions [9–11]. For example, *I. duncani* was included in an analysis by Tennant *et al*. [12] of changes in worldwide crocodyliform diversity patterns across the Jurassic/Cretaceous (J/K) boundary, with its preferred habitat recorded as ‘fluvial indet.’ based on previous interpretation of the entire Winton Formation being a fluvial/lacustrine unit (see Tennant *et al*. [12], electronic supplementary material, SI 2). Recent research has instead found a brackish water signature in the fossiliferous concretions from Isisford [6]. We propose that taphonomic analysis is required to clarify whether *I. duncani* inhabited this brackish water setting in life.

The degree of *I. duncani* fossil skeletal articulation, completeness, bone surface modification, presence of fractures, and proximity to other skeletal elements can indicate interference by biostratinomic agents, and whether each carcass underwent fluvial transport prior to burial. A complete and articulated skeleton is likely to represent a ‘rapidly’ buried, autochthonous carcass [13–17]. However, it may also represent an allochthonous carcass, transported a relatively long distance in sediment-laden floodwaters or turbidity flows [18,19]. Allochthonous material may also comprise isolated and abraded skeletal elements damaged during transport [14,20–23]. Bones with minor signs of abrasion, or those with tooth bite marks, scratches and acid etching, may have only been transported a short distance via fluvial action, or scavenging, respectively (parautochthonous) [24–27]. The fluvial transport potential of *I. duncani* skeletal elements, as for most vertebrates, would also have been controlled by bone morphology, size, density, and the presence or absence of soft tissues holding skeletal elements in articulation [17,20,22,28–33]. However, it is not known whether the *I. duncani* carcasses underwent ‘bloat and float’ [34–39], and therefore could have been transported long distances with minimal bone surface modification.

The majority of taphonomic classification schemes are typically based on shelly assemblages or mammalian decay patterns, and must be used in concert with empirical data from actualistic decay studies of analogous species. This is especially crucial in determining the influence of biostratinomic processes on *I. duncani* carcasses in the taphonomically active zone (TAZ) (after Powell *et al*. [40]). The stage of decay reached in the TAZ prior to burial can be deduced by comparing fossil skeletal articulation and completeness. Finally, the catalyst for concretion formation around *I. duncani* carcasses (with or without soft tissue) at Isisford has not been ascertained.

In order to clarify the above issues, in this study we documented the taphonomic history of the *Isisfordia duncani* holotypes, paratypes and referred specimens. We used a modified methodology from Beardmore *et al*. [41], Dodson [20] and Syme & Salisbury [42] to compare degrees of articulation and completeness between skeletal elements. We recorded the presence or absence of microwear and scratch marks on bone surfaces to determine whether bones were weathered, abraded during transport, scavenged or trampled prior to burial. These biostratinomic signatures were then compared to previously published taphonomic classification systems as well as observed decay patterns in juvenile *Crocodylus porosus* to determine the transport potential of skeletal elements and degree of autochthony. This hypothesized taphonomic history was then considered in light of the inferred upper and lower delta plain depositional environment of the Early Cretaceous of Isisford. We also consider the possibility of cadaver decomposition island (CDI) formation in the taphonomic history and the potential contribution to calcite cement precipitation and concretion formation. Finally, the results have implications for understanding fossil assemblages of the Winton Formation and other coeval deposits with similar depositional histories. The presence of crocodyliform fossils at this latitude also has bearing on inferred local palaeotemperatures.

## 2. Geological setting

The Winton Formation comprises volcanolithic sandstones, interbedded with mudstones and siltstones, in part calcareous, with minor intraformational conglomerates, lignites and coal deposits [43–49]. Palynological data and detrital zircon dating indicates the Winton Formation was deposited between the late Early Cretaceous (late Albian to early Cenomanian, approx. 103–100.5 Ma) and the early Late Cretaceous (late Cenomanian–early Turonian, approx. 93.9 Ma) (stratigraphic boundaries following Cohen *et al*. [50]) [51–57]. The upper Winton Formation has been interpreted as a freshwater alluvial plain deposit [43–45,53,58]. The presence of channel and overbank formations is indicative of ancient rivers that drained west and south toward the epicontinental Eromanga Sea [43,46,59,60]. As these rivers neared the ancient coastline, they deposited sediments in an upper to lower delta plain setting and formed the lower Winton Formation [7,53]. The volcanolithic component of the upper and lower Winton Formation most likely derived from rift-related volcanoes in the Whitsunday Volcanic Province to the east [7,53,61–64]. The rivers flowed out to sea in delta fans preserved in the marine to paralic Mackunda Formation. The Eromanga Sea went through its final regressive phase throughout the late Early Cretaceous to the early Late Cretaceous [44,52,57,65,66].

**Figure 1.**
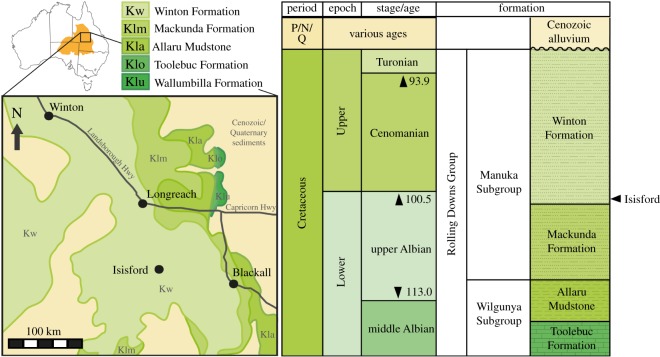
Map showing the Isisford site locality. The site is located near the town of Isisford, with minimal exposure but abundant *ex situ* concretions of Early to Late Cretaceous aged Winton Formation. The subsurface extent of the Winton Formation is shown as light green and labelled ‘Kw’; exposure at the surface is not as extensive but instead often obscured by modern and Cenozoic alluvium (see figure 2). The fossil material from Isisford is from the upper Albian portion of the Winton Formation. Although the Winton Formation conformably overlies the paralic/marine Mackunda Formation, Allaru Mudstone and Toolebuc Formation at Isisford, elsewhere to the west and north these paralic/marine units are coeval with the succession at Isisford.

The fossils from Isisford are found in *ex situ* indurated sandstone concretions of the lower Winton Formation (figure 1) [6,7,53]. The Winton Formation at Isisford has assigned a maximum age of 102.2–100.5 Ma, and is older and potentially closer to the contact with the conformably underlying Mackunda Formation (marked by the first appearance of coal beds) than the upper Winton Formation sites [6,7,53]. The shift up-section from the Mackunda Formation to the Winton Formation represents the progradation of a large delta system westward, resulting in a transition from a marine near shore and delta fan settings to lower and upper delta plain settings [7]. This confirms an earlier analysis of calcite cement oxygen and carbon stable isotopes that indicated the presence of mixed marine and fresh water in the pore waters of the lower Winton Formation [6]. The presence of a low-energy deltaic distributary channel facies in the lower Winton Formation at Isisford is also evidenced by mud rip-up clasts and wood debris preserved in fine-grained sandstones [6,7,43].

Today, floodplains and typically dry braided streams of the Barcoo River dominate the landscape surrounding Isisford. Summer rainfall resulting from severe thunderstorms and cyclonic activity can result in flash floods. Although this weathering can destroy exposed fossils across the Winton Formation, fossils found in concretions at Isisford seem to be protected from these forces; in fact, high levels of erosion in this area from flood events may remove the surrounding bedrock, and this in turn can aid in fossil discovery. Although the dynamic nature of the landscape may have transported these concretions from their original place of formation, they are all found in close proximity to one another and to concretion-like structures eroding out of outcrop. Where concretions have broken into separate portions, the pieces are typically found in close association such that the original concretion can usually be pieced back together. All of the concretions have a similar lithology to the Winton Formation in nearby outcrop and core samples [6,7,53]. These concretions and core samples both have similar mixed marine and freshwater stable isotope signatures [6]. Based on this evidence we therefore consider these concretions to have originated from the distal-fluvio-deltaic horizons within the lower Winton Formation at Isisford.

Macrofossils recovered from the Isisford locality concretions thus far include the aforementioned crocodyliform *I. duncani* Salisbury *et al*. 2006 and ichthyodectiform *Cladocyclus geddesi* Berrell *et al*. 2014, as well as other undescribed osteichthyans (including a halecomorph indet.), undescribed small-bodied non-avian dinosaurs and moulds of woody plant material [1,2,67,68]. Unlike the sauropod- and theropod-bearing fossil localities from the upper Winton Formation [69–84], the Isisford locality has not, to date, produced any large-bodied (greater than 5 m body length) non-avian dinosaur remains. Also conspicuous by their absence are lungfish and turtle fossils, having been recovered from the older and more coeval deposits of the marine Toolebuc Formation, Allaru Mudstone and Mackunda Formation [85–92] and the younger upper Winton Formation [52,89,90]. These absences could be a result of taphonomic bias or reflect the actual faunal composition at the time.

## 3. Material and methods

### 3.1. Field localities

**Figure 2.**
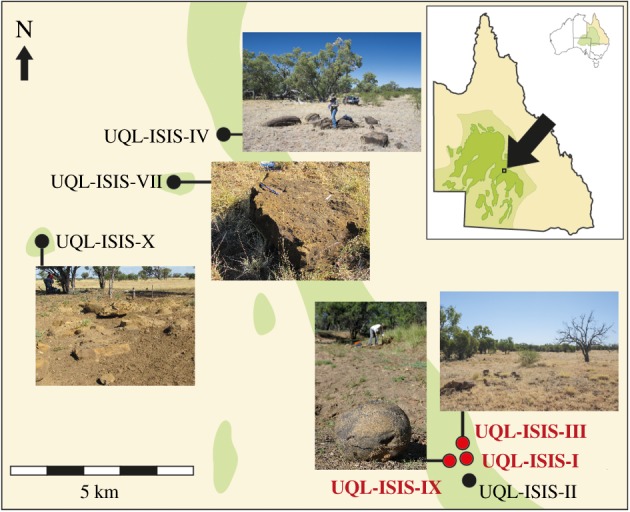
Sites at the Isisford fossil locality that bear fossil flora and/or fauna from the Winton Formation. *Isisfordia duncani* fossils have been found at UQL-ISIS-I, UQL-ISIS-III, and UQL-ISIS-IX, indicated in the figure by red and bolded font. Light green areas denote areas where *ex situ* concretions and outcrop of the Winton Formation occur, with light yellow indicating modern and Cenozoic alluvium. Modified from Syme *et al*. [6].

Of the seven vertebrate- and plant-fossil bearing sites at the Isisford locality, *I. duncani* fossils have been recovered from three—at University of Queensland Field Locality (UQL) ISIS-I, UQL-ISIS-III, and UQL-ISIS-IX—all within a 1 km^2^ area (figure 2). All the *I. duncani* fossils considered in this study were found in *ex situ* Winton Formation sandstone concretions.

### 3.2. Fossil material

SYSTEMATIC PALAEONTOLOGY

CROCODYLIFORMES Hay, 1930 [93]

MESOEUCROCODYLIA Whetstone & Whybrow, 1983 [94]

EUSUCHIA Huxley, 1875 [95]

*ISISFORDIA DUNCANI* Salisbury *et al*., 2006 [2]

#### 3.2.1. Holotype

**Figure 3.**
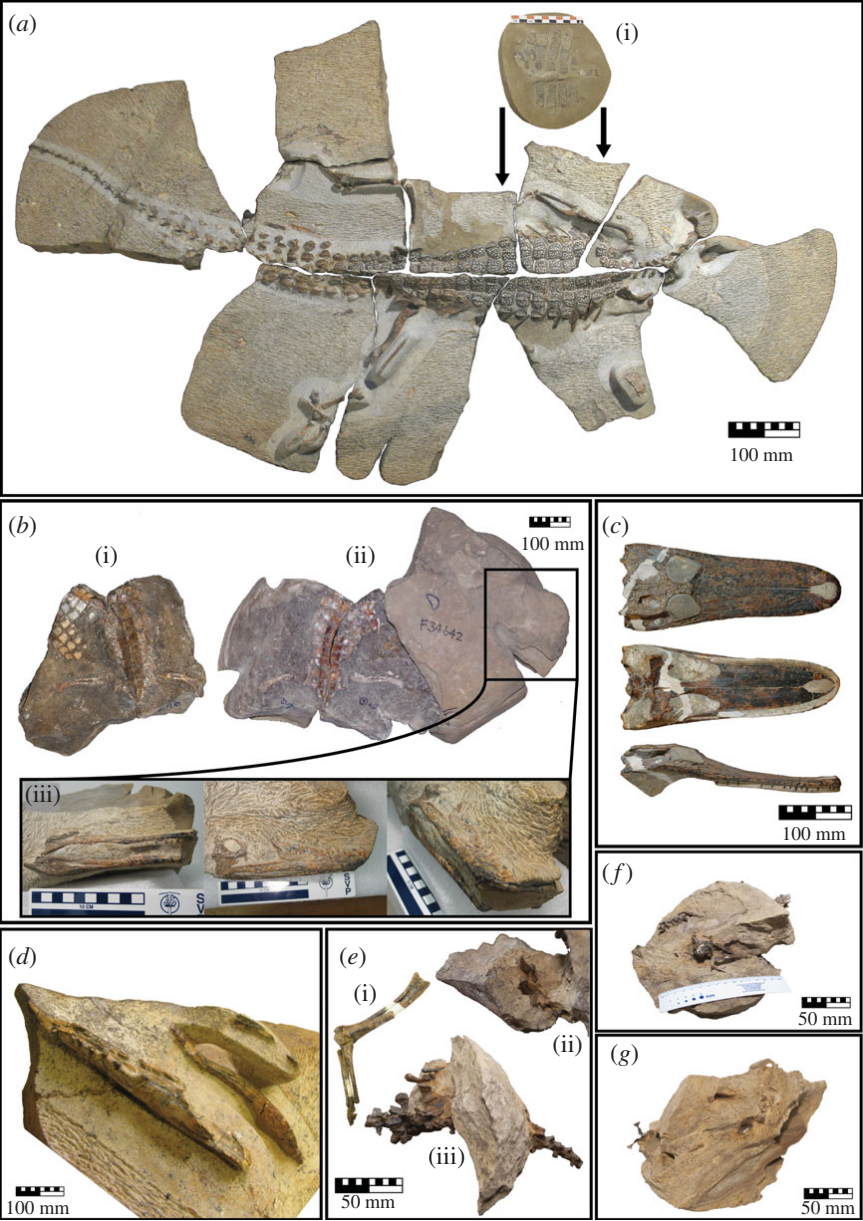
Images of all *I. duncani* specimens examined in this study. (*a*) QM F36211 (holotype), an articulated and near complete skeleton in dorsal aspect, (i) gastral shield in external (ventral) aspect, preserved in its *in vivo* position as indicated by arrows; (*b*) QM F34642 (paratype), an articulated skeleton with the mouth partially agape, (i) partial trunk, femora and gastral shield in dorsal aspect, (ii) counterpart to (i) showing trunk, femora and paravertebral shield in ventral aspect, (iii) articulated skull partially prepared in (from left to right) right lateral, dorsal, and right lateral/rostral aspect; (*c*) QM F44320 (paratype), a skull without mandible in (from top to bottom) dorsal, ventral and right lateral aspect; (*d*) QM F44319 (paratype), a partial mandible and part of a possible hyoid element in dorsal/left lateral aspect; (*e*) QM F58795, partially articulated and incomplete hindlimbs and caudal vertebrae of one individual, (i) partial left hindlimb shown in medial aspect, (ii) right pes showing right digit II with ungual rotated out of life position shown in lateral aspect (plantar surface facing up), (iii) right pes, vertebral elements and osteoderms preserved in their *in vivo* positions shown in left lateral aspect; (*f*) QM F58794, partially articulated juvenile remains including cranium, partial mandible, osteoderms, vertebrae, ilium, ischium and tibia; (*g*) QM F58793, disarticulated sub-adult or adult remains including partial crania.

QM F36211, a near fully complete and near fully articulated skeleton (figure 3*a*).

#### 3.2.2. Paratypes

QM F34642, a partially complete, articulated skeleton; QM F44319, a partial mandible and maxilla; QM F44320, a skull without the mandible.

#### 3.2.3. Referred specimens

QM F58793 and QM F58794 (provisionally assigned, currently under study), a sub-adult or adult and a juvenile; QM F58795 (provisionally assigned, currently under study), thoracic vertebrae, osteoderms, left and right pedal elements (figure 3*b*–*g*).

### 3.3. Taphonomic analysis

The numbers of skeletal elements present were catalogued, with the number of individual specimens (NISP), minimum number of elements (MNE) and minimum number of individuals (MNI) tallied. We used this information to calculate the degree of completeness of discrete skeletal units (§3.3.1) in comparison to the degree of skeletal articulation. Specimens were also examined for signs of weathering, abrasion, fracture, compression and bone surface modifications such as gnaw marks or trample marks. We recorded whether skeletal elements aligned with bedding planes (where observable) in the surrounding matrix. Finally, we estimated the transport potential of articulated elements or individual bones. The specific classification systems implemented are listed in the following sections.

#### 3.3.1. Degree of completeness

The degree of completeness was measured using the semi-quantitative methodology for fossil marine crocodylomorphs outlined by Beardmore *et al*. [41], with skeletal elements grouped as ‘units’ based on position in the body (such as cranial elements (head), cervical vertebrae (neck), thoracic and lumbar vertebrae (trunk), caudal vertebrae (tail), thoracic ribs (ribs), pectoral girdle with forelimbs (left and right forelimbs) and pelvic girdle with hindlimbs (left and right hindlimbs)). These skeletal units are then given scores ranging from 4 (fully articulated/complete) to 0 (no articulation/incomplete). On average, a score of 0 indicates 0–10% of skeletal elements are observed, 1 indicates 10–25% of skeletal elements within a given skeletal unit are observed, increasing in 25% increments up to and including category 4 indicating that 75–100% of skeletal elements are observed (see fig. 2 in Beardmore *et al*. [41] for more specific details pertaining to each skeletal unit). This classification system takes into account that skeletal elements may appear to be absent due to being concealed by matrix or other skeletal elements (denoted by Xh), or truncation at the edge of the matrix block, possibly due to post-diagenetic weathering having destroyed or removed elements (denoted by Xp) (following the criteria of Beardmore *et al*. [13,41]).

We opted to include a tenth and eleventh category for completeness of dorsal dermal skeleton (including the dorsal shield comprising a paravertebral shield and accessory osteoderms, and, where present, a separate nuchal shield) and the ventral dermal skeleton (including a gastral shield and separate gular shield, where present) respectively, as per the standard non-gavialoid eusuchian dermal skeleton [2,96]. In a complete *I. duncani* fossil, we would expect to see approximately 156 osteoderms in the dorsal dermal skeleton (including paravertebral, accessory, caudal and nuchal shield osteoderms) and 64 osteoderms in the ventral dermal skeleton (only the gastral shield is present). The score definitions for dermal skeletal units are shown in table 1. Beardmore *et al*. [41] conducted statistical analyses using these measures of articulation and completeness of fossils. By combining articulation scores with completeness scores, the values were then compared to trends outlined by Beardmore *et al*. [41], where Trend 1 characterizes high completeness with decreasing articulation, Trend 2 indicates decreasing completeness and articulation and Trend 3 characterizes a midway pattern between Trends 1 and 2. We chose to follow this methodology as it allows for semi-quantitative analysis of qualitative data.

**Table 1.** Definitions of dermal skeletal unit completeness and articulation scores. This table was used in conjunction with completeness and articulation scores outlined in fig. 2 of Beardmore *et al*. [41].

	dorsal and ventral dermal skeletal units	
score	completeness	articulation
0	between 0 and 10% of individual osteoderms present; equating to approximately 0–16 osteoderms of the dorsal dermal skeleton or approximately 0–6 osteoderms of the ventral dermal skeleton in *I. duncani*	score 0: between 0 and 10% of individual osteoderms preserved in their *in vivo* position or retaining interosteodermal articulations; very few to no osteoderms preserved in their *in vivo* position
1	limited completeness; 10–25% of individual osteoderms present; if present in life, one or more osteoderms with interosteodermal articulations absent; equating to approximately 16–39 osteoderms of the dorsal dermal skeleton or approximately 6–16 osteoderms of the ventral dermal skeleton in *I. duncani*	limited articulation; 10–25% of individual osteoderms preserved in their *in vivo* position; one or more osteoderms with interosteodermal articulations such as those in the gular shield or gastral shield (where present) disarticulated or rotated out of *in vivo* position
2	moderate completeness; 25–50% of individual osteoderms present; one or more paravertebral shield osteoderms and/or other osteoderms bound by muscles or ligaments absent; equating to approximately 39–78 osteoderms of the dorsal dermal skeleton or approximately 16–32 osteoderms of the ventral dermal skeleton in *I. duncani*	moderate articulation; 25–50% of individual osteoderms preserved in their *in vivo* position; one or more paravertebral shield osteoderms, caudal osteoderms, gastral shield osteoderms, and/or other osteoderms bound by muscles or ligaments disarticulated or rotated out of *in vivo* position
3	near full completeness; 50–75% of individual osteoderms present; one or more nuchal shield and/or other osteoderms residing in fatty tissues absent; equating to approximately 78–117 osteoderms of the dorsal dermal skeleton or approximately 32–48 osteoderms of the ventral dermal skeleton in *I. duncani*	near full articulation; 50–75% of individual osteoderms preserved in their *in vivo* position; one or more nuchal shield and/or other osteoderms residing in fatty tissues disarticulated or rotated out of *in vivo* position
4	almost full, to full completeness; 75–100% of individual osteoderms present; equating to approximately 117–156 osteoderms of the dorsal dermal skeleton or approximately 48–64 osteoderms of the ventral dermal skeleton in *I. duncani*	almost full, to full articulation; 75–100% of individual osteoderms preserved in their *in vivo* position and full retention of interosteodermal articulations (where present in life)

#### 3.3.2. Degree of articulation

We initially assessed the degree of articulation using a scheme developed for dinosaur fossil taphonomy by Dodson [20], who ranked remains from ‘A—complete or nearly so’ through to ‘K—incomplete articulated specimens—extent of original specimen unknown’ (see Table IV in Dodson [20]). This scheme assumes that absences of skeletal elements are true absences, and therefore does not account for loss of elements via erosion or collection bias [20]—while this works well for laterally extensive sedimentary units with fossils found *in situ*, the *ex situ* nature of the Isisford concretions coupled with skeletal element truncation at the edges of blocks means it is harder to determine if loss of elements represents true absence at the time of burial.

We decided to enhance our interpretations by using the Dodson [20] scheme alongside a second classification scheme created by Beardmore *et al*. [41] and modified by Syme & Salisbury [42]. This second scheme categorizes the articulation of skeletal elements within nine skeletal units: head (skull and mandible), neck (cervical vertebrae), trunk (prothoracic, thoracic, lumbar and sacral vertebrae), tail (caudal vertebrae), ribs (thoracic ribs), left forelimb with left pectoral girdle, right forelimb with right pectoral girdle, left hindlimb with left pelvic girdle portion and right hindlimb with right pelvic girdle portion. This scheme is hereafter referred to as intra-unit articulation, and uses a score for each skeletal unit from 4 to 0 (see fig. 2 in Beardmore *et al*. [41]). We also chose to include a tenth and eleventh unit for articulation of the dorsal dermal skeleton (including the dorsal shield comprising a paravertebral shield and accessory osteoderms, caudal osteoderms and, where present, a separate nuchal shield) and ventral dermal skeleton (including a gastral shield and separate gular shield, where present) respectively, as we propose that the articulation of osteoderms can indicate the presence and/or integrity of soft tissue at the time of burial. For example, the paravertebral shield comprises osteoderms imbedded in dermis, connected by interosteodermal ligaments, and attached to the underlying aponeuroses of the epaxial musculature and cingular ligaments [96–99]. Therefore, the disarticulation of the paravertebral shield would imply disruption or decay of dermis as well as this musculature and associated tendons and ligaments. Conversely, the nuchal shield of most eusuchians lies within dermis but is not attached to any ligaments or musculature [96,98,99]; therefore its disarticulation could only indicate disruption or decay of the dermis. Additionally, serrated sutures, or interosteodermal articulations, are present in some eusuchians [96,99]. As Syme & Salisbury [42] note, these could require greater degrees of decay to disarticulate, and may then detach in articulated segments. Definitions of the dermal skeletal articulation categories are shown in table 1. We reported average articulation scores for each *I. duncani* specimen both including and excluding these dermal osteoderm categories, to create a suitable database for comparison with other tetrapods either possessing or lacking dermal skeletons.

Finally, we used the classification scheme devised by Syme & Salisbury [42] to record the degree of articulation between the nine skeletal units described prior. This scheme has three categories: fully articulated (F)—maintenance of *in vivo* position, with no spaces or rotations between adjacent skeletal elements, partially articulated (P)—slight separation or rotation of adjacent skeletal elements and disarticulated (D)—separation of adjacent skeletal elements. This scheme is hereafter referred to as inter-unit articulation, recorded between the following units: head–neck (base of cranium to the atlas–axis complex), neck–trunk (cervical vertebra IX to (pro) thoracic vertebra I), trunk–tail (sacral vertebra II to caudal vertebra I), left and right forelimb–trunk (scapula and coracoid to (pro) thoracic vertebrae I and II), and left and right hindlimb–trunk (ilium to sacral vertebrae I and II). As the intra-unit articulation scheme already accounts for the degree of articulation between the ribs to trunk, an inter-unit articulation category for ribs–trunk was not required.

#### 3.3.3. Other taphonomic characteristics

The degree of weathering was recorded following the classification scheme used by Cook [100], Fiorillo [21], Prassack [101] and Ryan *et al*. [102], modified from Behrensmeyer [103] (table 2). Behrensmeyer [103] noted that weathering features on the surface of bones could differ between mammals of different body sizes as well as between different taxa, which have been accounted for in the Fiorillo [21] classification scheme. We feel that one feature of weathering Stage 1 [103], mosaic fracturing or cracking, needs further clarification. We identified ‘mosaic cracking’ as cracks on bone surfaces that did not penetrate cortical bone [101]. These mosaic cracks differ from ‘mosaic fractures’ and bone flaking that can penetrate the cortical bone (see fig. 10 in González Riga & Astini [113]).

**Table 2.** Taphonomic classification schemes used to describe the Isisford crocodyliform fossil material, after Cook [100], Fiorillo [21], Prassack [101], Ryan *et al*. [102], Behrensmeyer [103], Cook & Trueman [14], Haynes [104], Karr & Outram [105], Schmeisser McKean & Gillette [106], Njau & Blumenschine [27], Fernández-Jalvo & Andrews [107], Behrensmeyer *et al*. [108], Andrews & Cook [109], Andrews [110], Abdel-Maksoud & Abdel-Hady [111], Nicholson [112], Voorhies [28], Boaz & Behrensmeyer [29], Coard [17], and Coard & Dennell [33]. It should be noted that classification stages do not need to correspond between categories. These classification schemes were used in conjunction with table IV in Dodson [20].

	classification				
category	Stage 0	Stage 1	Stage 2	Stage 3	Stage 4
weathering stage (modified from Cook [100], Fiorillo [21], Prassack [101], Ryan *et al*. [102] and Behrensmeyer [103])	fresh bone (or tooth) with no sign of surface cracking or flaking	superficial cracks in the outer layers parallel to the bone fibres and do not penetrate cortical bone. Mosaic cracking may be present on articular surfaces	cracking and flaking or exfoliation of the outer layers. Cracks may penetrate bone cavities	outer layers of bone removed exposing cancellous tissue. Cracks penetrate bone cavities	
abrasion stage (modified from Fiorillo [21] and Cook & Trueman [14])	very angular: the bone is fresh and unabraded. Processes and bone edges are well-defined	subangular: the bone edges and processes are slightly abraded and polished	subrounded: the bone edges are well–rounded, processes are still recognizable. Moderate abrasion	rounded: edges show a high degree of rounding. Processes are remnant or absent. Heavily abraded	extremely rounded: bones often show a high degree of sphericity. Extremely abraded
fracture stage (modified from Cook & Trueman [14], Haynes [104], Karr & Outram [105] and Schmeisser McKean & Gillette [106])	bone/tooth is not broken	bone/tooth is broken in one or two places, not pervasive	bone/tooth is broken in many places, still recognizable	bone/tooth is highly fractured and may be unidentifiable	
compression stage [106]	bone/tooth is not compressed	bone/tooth is slightly compressed. Open cavities are slightly distorted. Solid areas display some distortion	bone/tooth is moderately compressed. Open cavities are highly distorted, solid areas are moderately distorted	bone/tooth is highly compressed. Open cavities are completely crushed, solid areas are heavily distorted	
PRESENCE/ABSENCE OF					
scavenging marks (following Njau & Blumenschine [27], Fernández-Jalvo & Andrews [107], and Andrews [110])	bite marks: partial or complete holes, pits, punctures				
	gnaw marks: sets of parallel grooves on bone – straight or curved, furrows, ragged edges, square-based grooves				
mosaic fractures (modified from Prassack [101] and Behrensmeyer [103])	cracks on bone surface that create a ‘mosaic-like’ pattern, and do not penetrate cortical bone				
trampling marks (following Fernández-Jalvo & Andrews [107], Behrensmeyer *et al*. [108] and Andrews & Cook [109])	closely spaced multiple parallel striations				
coloration/staining (following Abdel-Maksoud & Abdel-Hady [111], Behrensmeyer [103] and Nicholson [112])	variation in coloration of bone surfaces or matrix: mottling, concentric bands, gradational colour change				
alignment with substrate	bedding planes—cross beds, planar laminated beds, etc.				
	axial skeleton and/or dorsal surface of cranial elements alignment with bedding plane				
TRANSPORT POTENTIAL					
Voorhies transport potential (for disarticulated elements) (modified from Voorhies [28], Boaz & Behrensmeyer [29] and Cook & Trueman [14])	*group I*: Immediately transported by flotation or by saltation. In mammals this group typically includes the ribs, vertebrae, sacrum, sternum, and sometimes includes the scapula, phalanges and ulna.	*group II*: Transported later than Group I, usually by traction. In mammals this group typically includes the femur, tibia, humerus, metapodia, pelvis, radius, and sometimes includes the scapula, ramus, phalanges and ulna		*group III*: Resisted transport, lagging far behind other groups. In mammals this group typically includes the skull and mandible, and sometimes includes the ramus	
Coard transport potential [17,33]	*lag Group (disarticulated bones)*		*transport Group (disarticulated bones)*		
	dry bones: mandible, metapodials, vertebrae (atlas, axis), pelvis, femur, tibia, some podials and phalanges.		dry bones: cranium, vertebrae, ribs, sacrum, scapula, humerus, ulna, tarsals		
	wet bones: Mandible, atlas, axis, scapula, ribs, humerus, ulna, femur, tibia, metapodials, some podials and phalanges		wet bones: vertebrae, sacrum, pelvis, astragalus		
	*lag Group (articulated bones)*		*transport Group (articulated bones)*		
	dry bones: cranium and mandible (possibly), axis		dry bones: cranium and mandible, all vertebrae, atlas, ribs, sacrum, all front limb bones, all rear limb bones		
	wet bones: cranium and mandible (possibly), all rear limb bones, atlas		wet bones: cranium and mandible, all vertebrae, axis, ribs, sacrum, all front limb bones		

We also recorded other bone surface modifications such as the presence or absence of bite or gnawing marks in the form of pits, punctures, striations, furrows, ragged edges and square-based grooves, and the presence or absence of trampling in the form of shallow parallel striations (see examples in Njau & Blumenschine [27], Fernández-Jalvo & Andrews [107], Behrensmeyer *et al*. [108], Andrews & Cook [109] and Andrews [110]). Where possible, we noted whether skeletal elements were aligned perpendicular, parallel or oblique to bedding. The degree of abrasion for each element was categorized using the classification schemed adopted by Cook & Trueman [14] and Fiorillo [21]. Fracture patterns were also noted, where helical fractures indicated fresh bone, and fractures parallel or perpendicular to the bone shaft indicated fracture post-mineralization (following Cook & Trueman [14], Haynes [104] and Karr & Outram [105]). We also determined the degree of brittle or plastic compression of skeletal elements using a scheme developed by Schmeisser McKean & Gillette [106]. These are outlined in table 2.

The transport potential of individual skeletal elements in a fluvial environment was classified using a system modified from Voorhies [28], Boaz & Behrensmeyer [29] and Cook & Trueman [14], as outlined in table 2. As the Isisford specimens included articulated individuals, we also considered the transport potential of articulated skeletal elements (table 2). Both Coard [17] and Coard & Dennell [33] found that articulated skeletal segments and dry bones washed into water have a higher transport potential than disarticulated skeletal elements or wet bones. We followed the classification system devised by Coard [17] for mammalian bones that divides wet and dry bones into either a ‘Lag Group’ or a ‘Transport Group’. However, this classification scheme does not accommodate the fact that soft tissue can be attached to articulated skeletal elements.

Any discolorations or patterns of staining on both the bone and surrounding matrix were also noted (table 2). Staining can indicate presence of mineral-rich waters flowing around and through the bones, anaerobic bacterial activity or exposure to fire [103,111,112]. Bleaching could indicate prolonged exposure to UV radiation prior to burial [111], although prolonged UV exposure would typically present as weathering Stage 3 depending on mineralization. Mineralization also influences the colour of fossil bones, therefore the colours observed are considered to be more diagnostic of mineral species resulting from diagenesis. We nonetheless recorded colour variation for potential use in future taphonomic analyses.

## 4. Results

**Figure 4.**
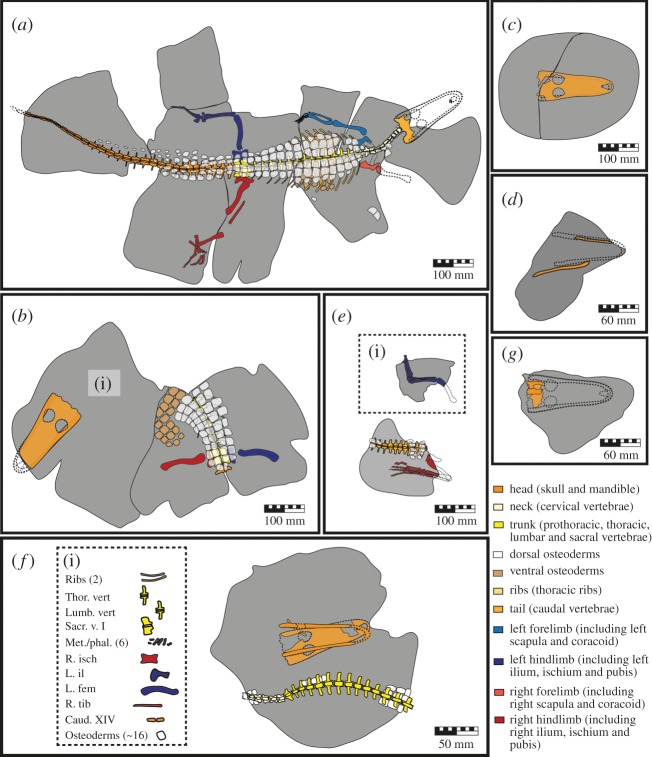
Diagram showing skeletal element orientation in concretion matrix (shown in grey) for each specimen of *I. duncani* previously shown in figure 3. All diagrams are drawn in dorsal aspect with respect to the orientation of cranial elements in the matrix (or vertebral elements where cranial elements are absent). (*a*) QM F36211 (holotype), near fully articulated and near complete adult. Of note is the near complete but partially disarticulated right hindlimb. The majority of paravertebral, accessory, caudal and ventral osteoderms are preserved in their *in vivo* position; (*b*) QM F34642 (paratype), probable adult with both femora preserved in their *in vivo* position relative to midline indicated by position of paravertebral osteoderms. Paravertebral and accessory osteoderms are in their *in vivo* positions, and gastral shield osteoderms have remained in articulation but rotated out of life position, (i) area of matrix proximal to the skull is still under preparation and may contain cervical vertebrae or forelimb elements preserved in their *in vivo* position; (*c*) QM F44320 (paratype), probable adult, complete cranium was not found in association with any other skeletal elements; (*d*) QM F44319 (paratype), probable adult, partially disarticulated as a portion of left mandible is still in occlusion with the left maxilla; (*e*) QM F58795, probable adult, partial right hindlimb preserved in its *in vivo* position relative to caudal vertebrae, (ii) partial left hindlimb is articulated and partially complete but position relative to the right hindlimb and caudal vertebrae was not recorded during preparation; (*f*) QM F58794, juvenile, cranium and mandible not preserved in their *in vivo* position, found in association with cervical and dorsal vertebrae, (i) numerous skeletal elements were found associated with the specimen but exact position in matrix was not able to be recorded during preparation; (*g*) QM F58793, sub-adult or adult, skull is incomplete but elements are preserved in their *in vivo* position.

**Figure 5.**
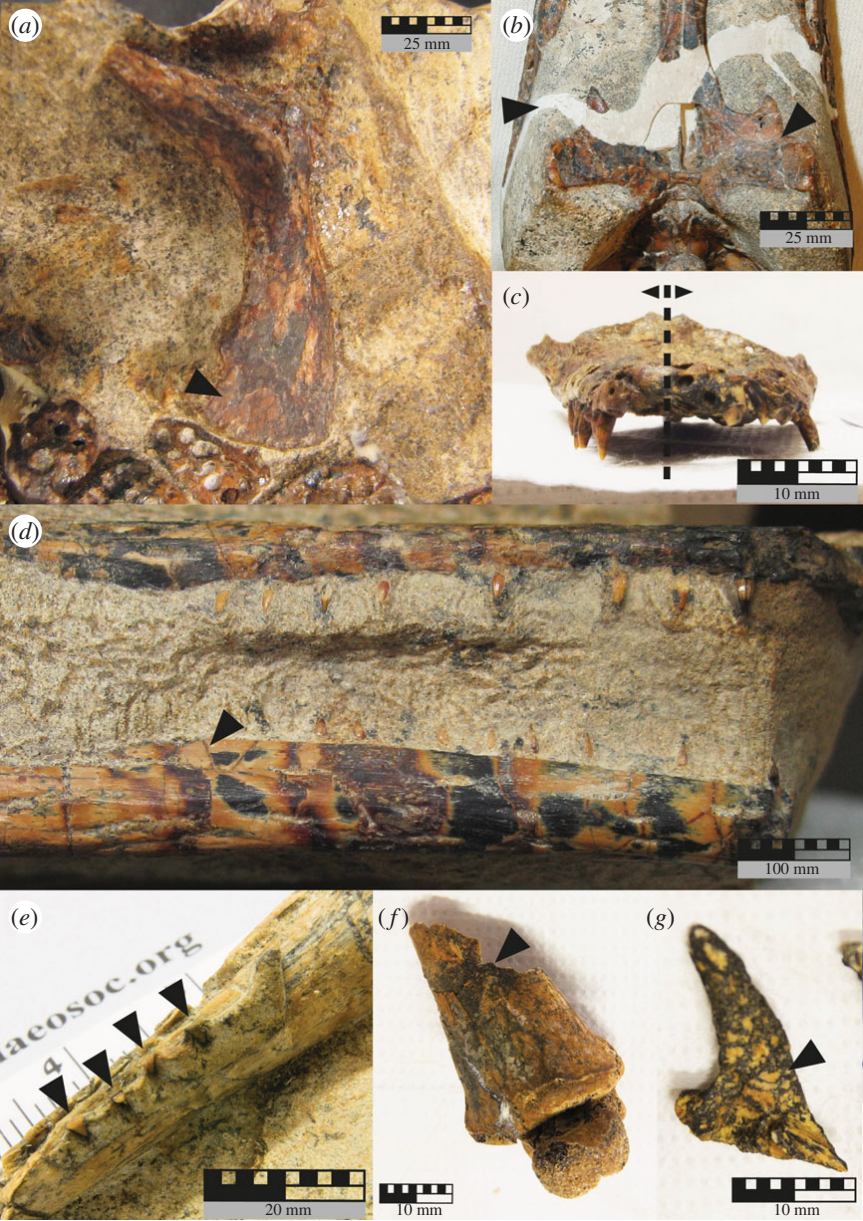
Photographs of taphonomic features observed in the seven *I. duncani* specimens. (*a*) QM F36211 (holotype) dorsal surface of the right scapula, arrow points to mosaic cracking, (*b*) QM F44320 (paratype) ventral surface of the basicranium, left arrow indicates resin infilling the diagonal fracture running from the right quadrate to the left jugal, right arrow indicates mosaic cracking and small fractures, (*c*) QM F58794 skull in rostral view, with black dashed line indicating midline of skull and arrows indicating the right and left halves of the skull: note the distortion of the left mandible, (*d*) QM F34642 (paratype) right maxilla and mandibular ramus in right lateral aspect, showing the separation of the jaws at the time of burial, black arrow indicating one of many mosaic cracks, (*e*) QM F44319 (paratype) left mandibular ramus in left lateral/dorsal view, arrows indicating apices of the maxillary teeth preserved in occlusion with mandible, (*f*) QM F58795 right cranial surface of the distal portion of right tibia and fibula (obscured) with calcaneum and astragalus in articulation, arrow indicates transverse/diagonal fracture, (*g*) QM F58793 left squamosal in ventral view, arrow indicates mosaic fractures and black mottling.

Based on the associated nature of the fossil skeletal elements, along with most fossils occurring in discrete concretionary bodies (excluding QM F58793 and QM F58794 which were differentiated based on size) we were able to assign bone fragments (number of individual specimens; NISP) and whole bones (minimum number of elements; MNE) to individual *I. duncani* specimens (table 3). The holotype QM F36211 is interpreted to be an adult (i.e. a mature individual based on ontogenetic characteristics of the skeleton) [2]. Based on comparisons with the holotype, the six remaining fossils are interpreted to include four probable adults (QM F34642, QM F44319, QM F44320, QM F58795), one adult/sub-adult (QM F58793) and one juvenile (QM F58794). Portions of articulated skeletal units are often found truncating the edges of concretions: for example, the right forelimb and rostral portion of the skull of the *I. duncani* holotype QM F36211 (figures 3*a* and 4*a*); the left femora, right femora and tibia/fibula of QM F58795 (figures 3*e*, 4*e* and 4*e*1); and the vertebral column of QM F58794 (figures 3*a* and 4*a*). We do not consider the absence of these elements to represent scavenging, pre-burial bone erosion or coincidental alignment with concretion boundaries. Rather, we consider it more likely that the now absent skeletal elements were present at the time of burial but (i) did not have concretion cement precipitate around them, and were then either not fossilized during diagenesis or were eroded during post-diagenetic deep weathering events, or (ii) were fossilized and are in unrecovered portions of concretions still residing at the fossil locality. Diagrams of skeletal material analysed are shown in figures 4 and 5, and all results of the taphonomic analysis are listed in tables 4 and 5.

**Table 3.** Summary of the *Isisfordia duncani* fossil elements recovered from the Isisford locality, including the number of identified specimens—where ‘specimens’ refers to the number of bone fragments—recovered per taxon (NISP), minimum number of elements (MNE) which may comprise multiple fragments each, and minimum number of individuals (MNI). We have included totals both without dermal skeletal units (as NISP and MNE), and with dermal skeletal units such as osteoderms included (as NISP inc. ost. and MNE inc. ost.). The relatively high NISP and MNE compared to MNI for *I. duncani* indicates that the majority of individuals comprise a multitude of articulated and/or closely associated skeletal elements. Approximate size range taken from and estimated using Salisbury *et al*. [2].

higher order taxon	genus and species	NISP	NISP inc. ost.	MNE	MNE inc. ost.	MNI	approx. body length range (m)
Crocodyliformes, Eusuchia	*Isisfordia duncani*	327	622	308	594	7	0.5–1.1

**Table 4.** Completeness (Cp.) and articulation (Art.) scores for *I. duncani* specimens from Isisford. As per Beardmore *et al*. [41], Xp denotes skeletal units that are not visible due to truncation with the edge of the block or blocks missing, and Xh denotes skeletal units obscured by matrix or overlying skeletal elements. Beardmore *et al*. [41] propose that specimens with three instances of either Xp or Xh in the dataset should be omitted from further analysis: in this instance, all specimens except the holotype QM F36211 were excluded from box plot and ‘Trend’ category assignation. The average completeness and articulation for QM F36211 was calculated both with and without the dermal skeletal units: with the dermal skeletal units, compared to a maximum total score of 36 (4 for each of the 9 of 11 units omitting Xp/Xh data); without the dermal skeletal units, compared to a maximum total score of 28 (4 for each of the 7 of 9 units omitting Xp/Xh data). The data were then compared with the trend lines proposed by Beardmore *et al*. [41] where possible.

field site	UQL-ISIS I				UQL-ISIS III						UQL-ISIS IX					
	*Isisfordia duncani* Salisbury *et al*. 2006															
specimen	holotype (QM F36211)		paratype (QM F34642)		paratype (QM F44319)		QM F58793		QM F58794		paratype (QM F44320)		QM F58795		average (nearest whole category)	
category	Cp.	Art.	Cp.	Art.	Cp.	Art.	Cp.	Art.	Cp.	Art.	Cp.	Art.	Cp.	Art.	Cp.	Art.
head	1Xp	0Xp	4	4	1	2	1	0	3	0	3	0	0Xp	0Xp	2	1
neck	4	4	0Xh	0Xh	0Xp	0Xp	0Xp	0Xp	4	4	0	0	0Xp	0Xp	1	1
trunk	4	4	2Xp	3Xp	0Xp	0Xp	0Xp	0Xp	4	3	0Xp	0Xp	0Xp	0Xp	1	1
ribs	4	4	2Xh	2Xh	0Xp	0Xp	0Xp	0Xp	1	0	0Xp	0Xp	0Xp	0Xp	1	1
dorsal osteo.	4	4	2Xp	2Xp	0Xp	0Xp	0Xp	0Xp	1Xp	1Xp	0Xp	0Xp	1Xp	1Xp	1	1
ventral osteo.	2	2	2Xh	2Xh	0Xp	0Xp	0Xp	0Xp	1Xp	0Xp	0Xp	0Xp	0Xp	0Xp	1	1
L. forelimb	4	3	0Xp	0Xp	0Xp	0Xp	0Xp	0Xp	0Xp	0Xp	0Xp	0Xp	0Xp	0Xp	1	0
R. forelimb	1Xp	1Xp	0Xp	0Xp	0Xp	0Xp	0Xp	0Xp	0Xp	0Xp	0Xp	0Xp	0Xp	0Xp	0	0
L. hindlimb	4	4	1Xp	1Xp	0Xp	0Xp	0Xp	0Xp	1	0Xh	0Xp	0Xp	3Xp	3Xp	1	1
R. hindlimb	4	2	1Xp	1Xp	0Xp	0Xp	0Xp	0Xp	1	0Xh	0Xp	0Xp	3Xp	4Xp	1	1
tail	4	4	0Xp	0Xp	0Xp	0Xp	0Xp	0Xp	1Xp	1Xp	0Xp	0Xp	2Xp	3Xp	1	1

**Table 5.** Table incorporating all other taphonomic classification systems used in this study of the *I. duncani* specimens from Isisford [14,17,21,27–29,33,100–112] that were used in conjunction with table IV in Dodson [20] and trends classified by Beardmore *et al*. [41]. We also chose to include counts of number of individual specimens (NISP) and minimum number of elements (MNE) with and without osteoderms, for ease of comparison in future with other tetrapods lacking dermal skeletons. The maximum number of skeletal elements in *I. duncani*, both excluding and including osteoderms, are listed next to MNE. Specimens QM F58793 and QM F58794 were found in close proximity, therefore NISP, MNE, and MNI are listed both grouped together (MNI = 2), and separately, to demonstrate the variation in NISP and MNE per individual. Where possible, life stage was determined by identifying closure of neurocentral sutures in vertebrae (closure proceeds caudally to cranially throughout ontogeny, with ‘adult’ defined as possessing closed neurocentral sutures in all vertebrae) and size comparison to the holotype that has been identified as an adult (see Salisbury *et al*. [2]). By comparing across specimen numbers, we determined whether disarticulated elements showed greater degrees of bone surface modifications. Degrees of articulation and completeness are the sum total of scores for each skeletal unit. The Dodson [20] classification scheme assumes absences are true absences; we selected the most likely class per specimen based on other taphonomic indicators (such as truncation with block, articulation versus completeness, and so on).

	*Isisfordia duncani* Salisbury *et al*. 2006						
specimen	holotype (QM F36211)	paratype (QM F34642)	paratype (QM F44319)	QM F58793	QM F58794	paratype (QM F44320)	QM F58795
field site	UQL-ISIS I		UQL-ISIS III			UQL-ISIS IX	
NISP	173	33	4	76		1	40
				4	72		
NISP (inc. osteoderms)	347	97	4	119		1	54
				4	115		
MNE (maximum 204 [2])	173	33	2	61		1	38
				2	59		
MNE (inc. osteoderms, maximum 424 [2])	347	97	2	95		1	52
				2	93		
MNI	1	1	1	2		1	1
				1	1		
life stage	adult	probable adult	probable adult	sub-adult or adult	juvenile	probable adult	probable adult
percentage completeness (not including osteoderms) (after Beardmore *et al*. [41])	84.8%	16.2%	2.0%	1.0%	28.9%	0.5%	19.6%
percentage completeness (including osteoderms)	81.8%	22.8%	0.9%	0.5%	21.9%	0.2%	12.7%
average completeness score (after Beardmore *et al*. [41])	3.3	1.1	0.1	0.1	1.7	0.3	0.9
average completeness score (including osteoderms)	3.3	1.3	0.1	0.1	1.5	0.3	0.8
average intra-unit articulation score (after Beardmore *et al*. [41])	2.8	1.2	0.2	0.0	0.9	0.0	1.1
average intra-unit articulation score (including osteoderms) (after Beardmore *et al*. [41])	2.8	1.4	0.2	0.0	0.8	0.0	1.0
average inter-unit articulation (F, P, D)^a^	F	F	D	D	P	D	F
weathering stage (modified from Cook [100], Fiorillo [21], Prassack [101], Ryan *et al*. [102] and Behrensmeyer [103])	Stage 1	Stage 2	Stage 1	Stage 1	Stage 1	Stage 1	Stage 1
abrasion stage (modified from Fiorillo [21] and Cook & Trueman [14])	Stage 0	Stage 0	Stage 0	Stage 0	Stage 0	Stage 0	Stage 0
fracture stage [106]	Stage 1	Stage 1	Stage 1	Stage 0	Stage 1	Stage 1	Stage 1
fracture types (modified from Cook & Trueman [14], Haynes [104], Karr & Outram [105] and Schmeisser McKean & Gillette [106])	transverse	transverse	transverse	nil	transverse/ diagonal	diagonal	transverse/ diagonal
compression stage [106]	Stage 0	Stage 0	Stage 0	Stage 0	Stage 1	Stage 0	Stage 0
scavenging marks (following Njau & Blumenschine [27], Fernández-Jalvo & Andrews [107] and Andrews [110])	nil	nil	nil	nil	nil	nil	nil
mosaic cracking (modified from Prassack [101] and Behrensmeyer [103])	present in all specimens. Black staining follows the mosaic crack lines.						
trampling marks (following Fernández-Jalvo & Andrews [107], Behrensmeyer *et al*. [108] and Andrews & Cook [109])	nil	nil	nil	nil	nil	nil	nil
bone coloration, staining (following Abdel-Maksoud & Abdel-Hady [111], Behrensmeyer [103] and Nicholson [112])	yellow-grey to light-dark brown with black mottling	yellow-light brown to red-dark brown with black mottling	yellow-grey to light-dark brown with black mottling	yellow-grey to light-dark brown with black mottling	yellow-grey to light-dark brown with black mottling	light brown to dark brown-red with black mottling	red-dark to light brown with black mottling
alignment with substrate	parallel with substrate, on one horizon	parallel with substrate, on one horizon	unclear due to method of preparation	parallel with substrate, spread vertically through multiple horizons	parallel with substrate, spread vertically through multiple horizons	unclear due to method of preparation	unclear due to method of preparation
Voorhies transport potential (modified from Voorhies [28], Boaz & Behrensmeyer [29] and Cook & Trueman [14])	Group I, II, III	Group I, II, III	Group III	Group III	Group I, II, III	Group III	Group I, II
Coard transport potential [17,33]	Lag and Transport Groups	Lag/Transport Group	Lag Group	---	Lag Group	---	Lag and Transport Group
decompositional class [20]	B—specimen complete or nearly so, some drifting of major elements	B—specimen complete or nearly so, some drifting of major elements	J—isolated bones	J—isolated bones	F—skull with incomplete articulated skeleton	I—skull without jaws	G—incomplete articulated skeleton
Trend [41]	Trend 3	n.a.	n.a.	n.a.	n.a.	n.a.	n.a.

^a^average across all skeletal units.

### 4.1. Taphonomic characteristics per specimen

#### 4.1.1. Holotype (QM F36211): near fully complete and near fully articulated skeleton

The holotype of *I. duncani*, QM F36211, comprises a near complete and near fully articulated skeleton with elements preserved in line with the dorsal plane (figures 3*a* and 4*a*; table 4). The concretion in which this specimen is preserved has fractured and separated into several discrete blocks. One such fracture divides the vertebral column parasagittally from approximately cervical vertebra V to caudal vertebra XII, with other fractures seemingly randomly distributed and approximately perpendicular to the vertebral column. Although the external basicranium is preserved, the rostral portion of the cranium and the mandible are missing. Two pairs of nuchal osteoderms are preserved, one dorsal and marginally laterally displaced from cervical vertebrae V and VI, and one approximately 105 mm caudolateral to the right scapula. Numerous paravertebral, accessory and caudal osteoderms, 141 in total, are preserved in their *in vivo* positions. Vertebral segments of thoracic ribs lie in their *in vivo* positions, underlying the paravertebral osteoderms. Approximately 33 ventral osteoderms forming part of the gastral shield are also preserved. Although the gastral shield was preserved in its *in vivo* position, it is depressed dorsally either side of the median plane, and has more ventrally positioned margins. No traces of mineralized dermal soft tissues were observed. The specimen is 84.8% complete excluding dermal skeletal units, and 81.8% complete when including dermal skeletal units (table 5). According to the Beardmore *et al*. [41] classification system, the holotype has an average completeness score of 3.3 regardless of whether we include dermal skeletal units.

The external basicranium is in articulation with the proatlas and axis. The cervical vertebrae are in articulation, curving gently to the left. One of the pairs of nuchal osteoderms is disarticulated, and has been displaced to a point that is approximately 105 mm caudolateral to the right scapula. The left scapula is dislocated. The left humerus is dislocated at the shoulder joint, but is in articulation with the radius and ulna. Some metacarpals and phalanges have been preserved proximal to the distal end of the radius and ulna. The left side of the body appears largely undisturbed, with elements in their *in vivo* articulations. The left forelimb is adducted, lying subparallel to the axial skeleton. The left femur is abducted and perpendicular to the axial skeleton and lies in its *in vivo* position (the dorsal surface facing up), with the left tibia, fibula, tarsals and metatarsals adducted and retracted to be parallel to the axial skeleton. The right scapula is disarticulated and displaced from the right coracoid, having moved marginally from its *in vivo* position (figure 5*a*). A small portion of the proximal articular surface of the right humerus truncates the edge of the block. The adjoining block potentially containing the remainder of the right forelimb could not be located in the field, but if preserved, this limb would most likely have lain adducted, subparallel or parallel to the axial skeleton with the forearm and manus extended (there is no other part of this limb in the adjoining cranial and caudal blocks) (figure 4*a*). The right hindlimb is abducted, being only slightly retracted at an 80-degree angle to the axial skeleton. Skeletal elements comprising the knee joint and the ankle joint are disarticulated; the right femur, tibia and phalanges have moved marginally out of their *in vivo* position, with the fibula and metatarsals I–IV showing a greater degree of disarticulation but remaining closely associated. The right femur is dislocated from the acetabulum, rotated with the dorsal surface facing up. The three right phalanges visible (I, II, and either III or IV) are preserved in association and are articulated. The average intra-unit articulation score is 2.8, whether excluding or including dermal skeletal units (table 5). The average inter-unit articulation category is F, indicating, on average, near full articulation of all present skeletal elements (table 5).

The minimal bone surface flaking observed suggests minimal to no weathering occurred, which therefore places this specimen in Stage 1 weathering (figure 5, table 5). The preserved portion of the skull is fractured along the median plane, and ‘transverse’ fractures are evident on all long bones (table 5). No abrasion of skeletal elements was observed (including trampling marks); therefore, we classify this specimen as having Stage 0 abrasion (table 5). No skeletal elements are distorted; therefore, the compression stage is classed as Stage 0 (table 5). Scavenging marks are absent. The skeletal elements are light brown to yellow in colour, with a black-grey mottled pattern covering all surfaces including the substrate, with black staining along the mosaic cracking present on bone surfaces (table 5; figure 5*a*). There appears to be no trace of mineralized soft tissues. The specimen is aligned parallel to the substrate, with all skeletal elements preserved with minimal vertical distribution (approx. 2 cm) on the same plane. The transport potential of individual skeletal elements spans Voorhies Groups I, II and III (table 5). The articulated segments can be classified as a combination of ‘Lag Group’ and ‘Transport Group’ (table 5). The overall decomposition class according to Dodson [20] is B (specimen complete or nearly so, some drifting of major elements). Using the Beardmore *et al*. [41] system, and including the dermal skeletal units, this specimen follows Trend 3, defined as a mixture of Trend 1 (representing a carcass that has predominantly decayed at the sediment–water interface) and Trend 2 (representing a loss of skeletal elements due to carcass transport during decay). When discounting the dermal skeletal units from analysis, the specimen plots within Trend 1.

#### 4.1.2. Paratype (QM F34642): partially complete, articulated skeleton

This paratype comprises a near complete cranium and mandible with the rostral portion of the rostrum weathered away, dorsal vertebrae VI to XV, sacral vertebrae I and II, caudal vertebra I, the left and right ilia and femora, approximately 14 thoracic ribs, approximately 45 paravertebral and accessory osteoderms forming part of the paravertebral shield, and approximately 19 gastral shield osteoderms (figures 3*b* and 4*b*; table 4). Some small bone fragments are present near-to and lying on the ventral surface of the mandible. The specimen is 16.2% complete excluding dermal skeletal units, and 22.8% complete including dermal skeletal units, which equates to average completeness scores of 1.1 and 1.3 respectively (table 5).

The cranium is on the same bedding plane as the exposed postcranial material, and neither has been rotated relative to the other. Therefore, although the neck and cranial portion of the trunk have not been exposed, the cranial part of the skeleton is in its *in vivo* position relative to the post-cranial material indicating a high degree of articulation at the time of burial. The mandible is in articulation with the cranium but with the jaws agape at an angle of approximately 20 degrees such that the rostral-most parts of the maxillary and mandibular rostra are 1.4 cm from each other (figure 5*d*). The paravertebral and accessory osteoderms are articulated and preserved in their *in vivo* position, while the gastral shield is articulated but displaced from its *in vivo* position. The left and right femora are in articulation with the left and right ilia respectively, with the dorsal surface of the right femora. Both the left and right femora are abducted and perpendicular to the axial skeleton (figures 3*b* and 4*b*). Given the *in vivo* positions and high degrees of articulation of the visible skeletal elements, we expect that further preparation of adjacent matrix will reveal cervical vertebrae, pectoral girdles and forelimbs in their *in vivo* positions. This specimen has a current average intra-unit articulation score of 1.2 excluding dermal skeletal units, but increases to 1.4 when including dermal skeletal units, and would likely increase further still with the exposure of more skeletal elements (table 5). The degree of inter-unit articulation of the few skeletal elements and osteoderms present is categorized as F—fully articulated (table 5).

Minor cracks/flaking of the exposed bone surface of the cranium and mandible can be seen (figure 5*d*). This is most similar to Stage 2 weathering, as there are cracks perpendicular to the bone fibre penetrating bone cavities (table 5). No fractures excluding mosaic cracking can be seen in the cranium or mandible (figure 5*d*). Excluding the post-diagenesis weathered surface of the rostral end, the cranial elements are unabraded (Stage 0 abrasion) (table 5) (this apparent inconsistency between weathering and abrasion stages is expanded on in the Discussion). The cranium and mandible are stained mottled black, dark red and yellow-brown (figure 5*d*). Some minor black staining can be seen in the matrix between the upper jaw and mandible (table 5, figure 5*d*). Staining is more prominent on the rostral two thirds of the upper jaw and mandible. Staining generally follows lines of bone surface mosaic cracking. This specimen includes skeletal elements that fall into Voorhies Groups I, II and III, and forms both Lag and Transport Groups (table 5). Using the Dodson [20] classification system this specimen represents class K (incomplete articulated specimens—extent of original specimen unknown). The Beardmore *et al*. [41] categorization omits specimens with more than three skeletal units missing from analyses; therefore this specimen was not assigned a trend category.

#### 4.1.3. Paratype (QM F44320): skull without mandible

This specimen comprises an isolated near complete skull without the mandible, with the only loss of skull material resulting from a fracture running obliquely across the basicranium (figures 3*c* and 4*c*; table 4). As the specimen comprises only a skull, the overall completeness is 0.5%, or 0.2% if the absence of the dermal skeleton is included, with a completeness score of 0.3 (table 5).

The skull is not in articulation with a mandible; therefore, the designated articulation score is 0, and the lack of any cervical material resulted in an inter-unit articulation classification of D—disarticulated (table 5).

The skull is unabraded, and most closely aligns with the weathering category Stage 1—although it does exhibit mosaic cracking and minor fractures of the lamellar bone on the dorsal, lateral and ventral surfaces (figure 5*c*), it is not apparent on the articular surfaces at the base of the skull (table 5). A diagonal fracture runs from the left jugal, through the left quadrate and parietal, to the right squamosal, and then continues through the right exoccipital and right quadrate (table 5, figure 5*c*). There is no evidence of compression, nor is there evidence of scavenging or trampling of the elements (table 5). The skull is classified as Voorhies Group III (resisted transport, lagging far behind other groups) (table 5). The specimen most closely aligns to both decompositional classes I (skulls without jaws) and J (isolated bones) (table 5), as it does not truncate the edge of the block and therefore appears to represent a true isolated element. The specimen is light brown with patches of red-dark brown and black mottling, and black infilling mosaic cracking boundaries (table 5; figures 4*c* and 5*c*). The Beardmore *et al*. [41] criteria omit specimens with more than three skeletal units missing from analyses; therefore this specimen was not assigned a trend category.

#### 4.1.4. Paratype (QM F44319): partial mandible and maxilla

This specimen comprises a partial mandible and part of a possible hyoid element, with the apices of teeth from the left maxilla preserved in occlusion with part of the left mandibular ramus (figures 3*d* and 4*d*; table 4). The remainder of the skeleton has not been found at the site (whether this is due to pre- or post-diagenetic taphonomic bias is unclear); therefore, the specimen is only 2.0% complete or 0.9% complete when including dermal skeletal units (no osteoderms were found at the site), and has an average completeness score of 0.1 (table 5).

The preserved portion of the right mandibular ramus is disarticulated and displaced relative to the position of the left mandibular ramus and the part of a possible hyoid element. As the apical portion of each of the left maxillary teeth is in occlusion with the left mandibular ramus (although the left maxilla was not preserved), we scored an average intra-unit articulation of 0.2 (figure 5*e*, table 5). The absence of cervical vertebrae means the inter-unit articulation is classified as D—disarticulated (table 5).

As we observed a mosaic-cracking pattern on lamellar bone that did not penetrate the cortical bone, we classified this specimen as belonging in weathering Stage 1 (table 5). Transverse fractures are randomly distributed across the specimen, and there are no scavenging or trampling marks (table 5). The specimen is not compressed. The specimen is yellow-grey to light brown-dark brown with areas of black mottling, and black infilling of mosaic crack and fracture lines (figure 5*e*, table 5). The transport potential of this specimen is classified as Voorhies Group III (resisted transport, lagging far behind other groups), and Lag Group (table 5). Due to the truncation of this specimen with the edge of the concretion, the decompositional class is a combination of H (skull with jaws) and J (isolated bones) (table 5). The Beardmore *et al*. [41] methodology omits specimens with more than three skeletal units missing from analyses; therefore this specimen was not assigned a trend category.

#### 4.1.5. Provisionally referred specimen QM F58795: caudal vertebrae segment, osteoderms, left and right pedes

This specimen comprises a segment of ten caudal vertebrae (II to XI) with approximately 14 caudal osteoderms, a partial left hindlimb including the distal-most quarter of the femur, tibia, fibula, astragalus, calcaneum, two distal tarsals, metatarsals V, three metatarsals and two phalanges, and a partial right hindlimb including a partial right ischium, the proximal end of the femur, distal ends of the tibia and fibula, astragalus, calcaneum, two distal tarsals, metatarsals I–V and nine phalanges (figures 3*e* and 4*e*; table 4). The specimen is 19.6% complete excluding dermal skeletal units from the total, and 12.7% including dermal skeletal units in the total, giving average completeness scores of 0.9 and 0.8 respectively (table 5).

All elements preserved show high degrees of articulation within each skeletal unit and between skeletal units. The caudal vertebrae are articulated and the caudal osteoderms are in their *in vivo* positions, lying dorsal of the vertebrae. The right hindlimb is articulated and preserved in its *in vivo* position proximal to the pelvic girdle. The ungual on right digit II is displaced laterally approximately 2 mm to the right from the articular surface of the third phalanx. Although the proximal end of the right femur is preserved in its *in vivo* position, adducted and near parallel to the axial skeleton, the distal portion of the femur and proximal portions of the tibia and fibula are not preserved. However, the right pes is adducted and retracted to lie parallel to the axial skeleton and the proximal portion of the femur, and the right ankle joint is intact (figures 4*e* and 5*f*). This orientation could be achieved without disarticulation at the right knee joint. Therefore, we propose that the entire right hindlimb was articulated and preserved in its *in vivo* position at the time of burial. The preserved left hindlimb comprises the distal portion of the femur in articulation with the tibia, fibula and left pes. The left crus is partially extended relative to the left femur, and the left pes is flexed relative to the crus. Both the knee joint and ankle joint are in their *in vivo* articulation. The left hindlimb was found in close association with the caudal vertebrae and right hindlimb; however, its exact position could not be recorded during fossil preparation. We have denoted this in figure 4*e*, but the high degree of articulation observed and proximity to the remainder of the skeleton indicate that the left hindlimb was likely preserved in its *in vivo* position, and the proximal articular surface of the femur was in articulation with the coxae (hip) at the time of burial. The overall intra-articulation is scored as either 1.1 (excluding dermal skeletal units) or 1.0 (including dermal skeletal units) (table 5), which appear artificially low due to obscured and/or absent skeletal elements as well as the unknown position of the left hindlimb. This is somewhat countered by the inter-articulation category of F—fully articulated (table 5).

Mosaic cracking is present on most bone surfaces including the distal-most phalanges; therefore the specimen is categorized as being in weathering Stage 1 (figure 5*f*, table 5). Transverse fractures are present across all elements, and a diagonal fracture is present through the shaft of the right femur and the right tibia and fibula (figure 5*f*), but no signs of abrasion are apparent excluding modern weathering of the right calcaneum and astragalus (figure 5*f*, table 5). The left phalanx has a helical fracture above the articulation with the left metatarsal (table 5). Multiple transverse fractures were observed along the left tibia, fibula and tarsals (table 5). The specimen does not show any signs of compression (table 5). There are no traces on bone surfaces of scavenging or trampling (table 5). The bones are stained a red-dark brown to light brown colour with black mottling following mosaic cracks (figure 5*f*, table 5). The specimen comprises elements in both Voorhies Groups I and II, and both the Lag and Transport Group (table 5). Given the truncation of elements with the edge of the block, this specimen represents the Dodson [20] class K (incomplete articulated specimens—extent of original specimen unknown). The Beardmore *et al*. [41] criteria omit specimens with more than three skeletal units missing from analyses; therefore this specimen was not assigned a trend category.

#### 4.1.6. Provisionally referred specimens (QM F58793 and QM F58794): partial cranial elements and partial semi-articulated skeleton, respectively

There are at least two individuals preserved in the same concretionary body as evidenced by a near complete skull and mandible (QM F58794; figures 3*f*, 4*f* and 5*c*, table 4) alongside isolated cranial elements including the pterygoid, parietal and left squamosal of a second, larger individual (QM F58793; figures 3*g*, 4*g* and 5*g*, table 4) alongside. The remainder of the postcranial skeletal elements recovered are more similar in size to the first individual (QM F58794), and include cervical (IV–IX), thoracic and lumbar (I–XV), and sacral (I) vertebrae, a left ischium and a right ilium, a complete left femora, a complete right tibia, as well as ribs, terminal caudal vertebrae, phalanges, and approximately 34 paravertebral and accessory osteoderms (figure 4*f*). The completeness of QM F58793 is 1.0%, or 0.5% when including dermal skeletal units (no osteoderms were found associated with this specimen), with an average completeness score of 0.1 (table 5). In contrast, QM F58794 is much more complete with 28.9% of skeletal elements visible, giving an average completeness score of 1.7; when dermal skeletal units are included, the overall completeness percentage and completeness score decreases to 21.9% and 1.5 respectively (table 5).

The pterygoid, parietal and left squamosal of QM F58793 are fragmentary and disarticulated resulting in an intra-unit articulation score of 0 (figure 5*g*, table 5). The inter-unit articulation was classified as D—disarticulated as no cervical elements were found in articulation or associated with the specimen (table 5). While QM F58794 possesses an articulated series of cervical, dorsal and sacral vertebrae (cervicals IV–IX, dorsals I–XV, and sacral I) with articulated paravertebral and accessory osteoderms, the skull is disarticulated, laterally displaced and parallel to the axial skeleton (figures 3*f* and 4*f*). The mandible is also disarticulated and lies on the dorsal surface of the skull. Of the paravertebral and accessory osteoderms, at least 18 are in their *in vivo* positions. While we could not ascertain the precise position of other postcranial elements due to the way the specimen was prepared (skeletal elements disassociating from each other during acetic acid dissolution of concretion cement), it appears that overall this specimen consists of segments of articulated skeletal elements that were displaced from one another prior to burial. This has resulted in a relatively low intra-unit articulation score of 0.9, or 0.8 if dermal skeletal units are included, and the most likely inter-unit articulation category for each skeletal element at the time of burial is P—partially articulated (table 5).

Both QM F58793 and QM F58794 show a mosaic pattern of superficial cracks, stained black, resulting in its categorization into Stage 1 weathering (figure 5*c*,*g*, table 5). Transverse fractures are also common among the QM F58794 long bones, with fracture lines stained black (table 5). QM F58794 possesses a diagonal fracture across the back of the basicranium (table 5). QM F58794 is the only *I. duncani* specimen recovered thus far to show any sign of compression, in the form of dorsoventral compression of the rostrum (figure 5*c*, table 5). Both specimens show no signs of abrasion, trampling or scavenging traces. Skeletal elements lie parallel to bedding planes, where visible, with elements spread vertically through the sediment. The skeletal elements are yellow-grey to light brown with patches of dark brown and black mottling, often following mosaic cracking (figure 5*c*,*g*, table 5). While QM F58793 is classified as Voorhies Group III (resisted transport, lagging far behind other groups), the multiple elements of QM F58794 span Voorhies Groups I, II and III (table 5). The skull elements of QM F58793 could not be classified using the Coard transport potential categories; however, QM F58794 is classed as the Lag Group category (table 5). The decompositional class for QM F58793 is a combination of I (skull without jaws) and J (isolated bones)—the specimen does not truncate the edge of the block, so the absence of other associated skeletal elements represents true absence (table 5). The decompositional class for QM F58794 is K (incomplete articulated specimens—extent of original specimen unknown) (table 5). The Beardmore *et al*. [41] criteria omit specimens with more than three skeletal units missing from analyses; therefore this specimen was not assigned a trend category.

## 5. Discussion

The majority of *I. duncani* fossils are articulated or semi-articulated with no apparent soft tissue preservation. The *I. duncani* specimens recovered from Isisford represent individuals that underwent partial decay and disarticulation in the TAZ prior to burial and fossilization. The residence time at the TAZ is classified using the five stages of vertebrate decay—(1) fresh, (2) bloated (when floating often begins in aquatic settings), (3) active decay, (4) advanced decay and (5) remains [42,114–116]. We have detailed the likely taphonomic pathways for each specimen in the following sections.

### 5.1. Taphonomic interpretation for each specimen

#### 5.1.1. Holotype (QM F36211): near fully complete and near fully articulated skeleton

As QM F36211 is mostly articulated, and no major loss or disruption of skeletal elements has occurred, this indicates that macroscavenging of the carcass did not take place. Microscavenging of soft tissues could have occurred without leaving a distinctive taphonomic trace. However, in extant juvenile *C. porosus*, microscavenging by insects is centred on orifices such as the cloaca resulting in disarticulation of pelvic girdle elements [42]. Given that the visible pelvic girdle elements of QM F36211 are still in their *in vivo* position, it appears that microscavenging (at least around the cloaca) did not take place. Microscavengers may have been precluded from orifices due to either submersion of the carcass in water, or burial of the carcass before it could float to the surface.

The external basicranium is in articulation with the proatlas and axis, therefore we propose that the cranium (and presumably the mandible) was likely still attached to the axial skeleton through tendinous and ligamentous connections to the occiput and via the dermis at the time of burial. The rostral portion of the cranium is absent, most likely because of exposure to post-fossilization, sub-aerial weathering processes, or that the portion of concretion containing the rostrum was not recovered from the field locality. Using the methodology outlined by Beardmore *et al*. [41], we created paired appendage plots to compare the degree of articulation and completeness between limb units. Taphonomic theory dictates that in animals with bilateral symmetry, decay should progress equally on either side of the symmetrical divide [13]. This specimen was the only one from Isisford to display asymmetrical patterns of completeness between the left and right forelimbs, and asymmetrical patterns of articulation between the left and right forelimbs and hindlimbs (although calculations of right forelimb articulation was hindered by lack of completeness) (see table 4, figures 4*a* and 5*a*, and the following paragraph). The right scapula has moved only marginally from its *in vivo* position, which indicates that the muscles, ligaments and other connective tissues holding it in position in life had decayed enough to allow its dislocation, but not enough for disarticulation (figure 5*a*). As the absence of the right forelimb elements also coincides with what we consider a clearly missing portion of the original concretion (figure 3*a*), it is more likely that the forearm was present at the time of burial and originally fossilized in association with the rest of skeleton, and then upon exposure of the fossil was eroded or broken and separated from the skeleton.

The right hindlimb more closely resembles the advanced stage of decay whereas the remainder of the body resembles a carcass in the fresh or active decay stage (figure 3*a*). If decay had occurred as the carcass floated in a body of water deeper than approximately three times the dorsoventral height of the individual, we would expect to see higher degrees of disarticulation in all limbs and the axial skeleton [42]. However, bloat and float in shallow water would limit the vertical space for gravitational forces to disarticulate skeletal elements symmetrically across the body. Disarticulation of the right hindlimb alone may have occurred if the dermis and underlying tissues were disrupted via an injury or pathology. This would cause decay to progress more rapidly in this area, although there is no bone surface abrasion or wound pathology to support this supposition. We instead propose that the entire carcass excluding the right hindlimb was buried during the fresh or active decay stage. While the rest of the carcass decayed underground, the right hindlimb decayed at the sediment–water interface. The hindlimb was then subject to further aerial or aqueous weathering, minor water current movement, and potentially microscavenging. This resulted in the further disarticulation of exposed skeletal elements, before they were finally buried during the advanced decay stage.

All the osteoderms with the exception of those from the nuchal shield are still in their *in vivo* positions. In life, the osteoderms of the paravertebral shield in the trunk and tail base would likely have been tightly integrated with the underlying aponeuroses of the epaxial musculature; the accessory osteoderms and those in the remainder of the tail sit in the dermis, as has been observed in extant eusuchians [96,97]. The accessory osteoderms are also likely to be have been connected to the paravertebral shield via interosteodermal ligaments and sutural joints. Although there is no perceptible trace of soft tissue preservation, the dermis inclusive of the osteoderms must have been intact at least prior to or during burial to hold these osteoderms in their *in vivo* position. Conversely, one of the transverse pairs of nuchal osteoderms is not in its *in vivo* position but is still closely associated with the rest of the skeleton. In life, nuchal osteoderms of non-gavialoid eusuchians are not integrated with the epaxial muscles and sit in the dermis, often external to fatty tissue [98]. The fatty tissue and dermis must have decayed enough to allow for these osteoderms to be transported a short distance from the carcass. This may indicate that, at least in the region of the neck, adipocere did not form rapidly enough to keep this osteoderm pair in place.

The depression of the articulated gastral shield either side of the midline indicates that the carcass was buried ventral surface up—the weight of overlying sediment and decay of internal organs caused the dermis to collapse around the vertebral column. The rotation of carcasses along their long axis to a ventral-surface-up orientation during bloat and float has been observed for carcasses of crocodilians [42,117] as well as other vertebrates including fetal pigs [118], coots [119] and fish [37,120]. The disarticulation of the nuchal shield prior to burial, along with minimal disarticulation across the body, seems to be at odds with this interpretation of a carcass decaying while lying with its dorsal surface in contact with the substrate. However, we propose that the carcass may have been either only slightly buoyed up by decay gasses, or bloated and floated in relatively shallow water, enough to raise the dorsal surface of the neck off the substrate and allow for the nuchal shield to detach but remain associated with the carcass once it settled on the substrate. Under this scenario, most of the carcass was then buried during active decay before further disarticulation could occur, with the right hindlimb remaining exposed until advanced decay, after which time it too was buried.

The specimen was initially assessed using Dodson [20] as belonging to decomposition class K. However, as we propose that the absence of skeletal elements (right forelimb, rostral portion of skull) does not represent true absence, the decompositional class most suited to this specimen is B (specimen complete or nearly so, some drifting of major elements). The inferred decay history for class B as proposed by Dodson [20], ‘A period of subaqueous exposure in low energy environment before burial. Decomposition has begun on extremities’, also aligns with our taphonomic interpretation. This specimen falls into Trend 3, which is defined by Beardmore *et al*. [41] as a combination of Trends 1 and 2: decay with no transport, or bloat and float with elements lost during transportation of carcass, respectively. If the dermal skeleton is not included in the assessment, this specimen plots closer to Trend 1. We propose that decay with no transport and therefore no loss of elements is the most likely taphonomic pathway and that this specimen is autochthonous.

As the remains were buried in a deltaic setting [6,7], and transportation of the carcass did not occur, we propose that this individual decayed either in the shallow waters of the delta, or in a crevasse splay or overbank deposit nearby. The skeletal elements lie mostly preserved in their *in vivo* position aligned parallel to the dorsal plane, with no elements dipping or rising through the matrix along the horizontal plane. The disarticulated osteoderms found associated with the skeleton did not leave a perceptible transportation path scoured into the sediment. Behrensmeyer [22] has calculated the transport potential of a modern species of *Crocodylus* dermal osteoderm as equivalent to a 3.1 mm quartz grain; as the *I. duncani* holotype concretion matrix consists of fine to medium sized quartz grains and feldspatholithics (125–500 µm), we propose that depositional currents were not able to transport any grains or elements larger than 125–500 µm. Mud rip-up clasts and other signs of periodic flooding events coupled with deposition of sediments in quiet-water conditions are present in the lower Winton Formation at Isisford [6]. This periodic flooding may have allowed for the *I. duncani* holotype to be partially covered by sediment over multiple burial events with minimal transport of disarticulated elements before complete burial.

#### 5.1.2. Paratype (QM F34642): partially complete, articulated skeleton

Similar to the *I. duncani* holotype, *I. duncani* QM F34642 would have partially decayed prior to burial and was in all probability not disturbed by macroscavengers. There are no other traces of scavenging marks on the remaining skeletal elements. It is more likely that the absent hindlimbs are either currently obscured by matrix, or were not able to be located in the field. As the absence of these elements may not represent true absence, it is likely that this specimen more closely aligns with the Dodson [20] decomposition class B (specimen complete or nearly so, some drifting of major elements) and the inferred decay history of, ‘a period of subaqueous exposure in low energy environment before burial; decomposition has begun on extremities’. Both the dorsal (paravertebral, accessory and caudal) and gastral osteoderms are articulated, suggesting the presence of undecayed connective tissues at the time of burial. Although the articulated portion of gastral osteoderms is displaced from its *in vivo* position, the presence of sutured margins coupled with intact interosteodermal ligaments would allow for retention of articulation prior to burial. There are no other signs of abrasion and there is minimal weathering of the skeletal elements. As the specimen contains elements that are easily removed due to current action, combined with the presence of articulated osteoderms, we propose that at least some soft tissues were still in place at the time of burial, and that the carcass did not float in nor sink through water of a depth greater than three times the dorsoventral height of the individual (given the results of Syme & Salisbury [42]).

As the mandible is articulated with the skull, but is not in occlusion with the upper jaw, the mouth must have been agape during burial and filled with sediment (figure 5*d*). During a decay experiment using juvenile *C. porosus* by Syme & Salisbury [42], the jaws of each individual did not open until after each carcass began floating approximately 4 days post-mortem, excluding one individual (CR2B) whose mouth opened approximately 10 degrees/2 cm two days post-mortem and prior to floating. The carcasses buried immediately post-mortem (Treatment 1) both had their mandibles in occlusion with their upper jaws. Similarly, the carcasses that floated prior to burial (Treatment 2) also had their mandibles in occlusion with their upper jaws. This indicates that, at least in juvenile *C. porosus*, for a carcass to be buried with sediment filling its oral cavity, burial must occur a few days post-mortem after bloat gasses have been generated but before the carcass floats [42]. However, other mechanisms that inhibit floating could increase the timeframe in which the carcass may be buried. We propose that at least the rostral portion of the carcass was buried during the bloat phase, during which time the jaws remained open and filled with sediment. For the disarticulation of the gastral osteoderms to occur with minimal weathering of other skeletal elements, the trunk must have remained unburied during the active decay stage. Burial in the shallow waters of a deltaic distributary channel or overbank deposit during flood events could result in these taphonomic characteristics. We conclude that the carcass did not float, or floated only in shallow water, prior to burial and was not transported far or at all from its original place of death, and is therefore classed as autochthonous.

#### 5.1.3. Paratype (QM F44320): skull without mandible

As this specimen consists only of an isolated and disarticulated but complete cranium, we propose that this individual's carcass bloated and floated, with the skull disarticulating during transport or as the carcass sank and reached the sediment–water interface. The lack of tooth marks or spiral bone fractures on the cranium indicates that it was not separated from the post-crania by a predator or scavengers. We consider the absence of the mandible and post-crania to represent true absence, as the cranium does not truncate the edge of the block and therefore was an isolated skeletal element at the time of burial. Skulls generally resist fluvial transport (Voorhies Group III) and as this specimen shows no signs of abrasion and minimal weathering, it is likely the skull detached from a floating, decaying carcass (either during the bloat stage or active decay stage). It then came to rest on the substrate and was subsequently buried either at or near to the original place of death. The assigned Dodson [20] class of I aligns with this interpretation, with an inferred decay history of, ‘early stage decomposition, carcass floating in channel before burial’—class J was also assigned, but as the specimen was assigned to Voorhies Group III coupled with minimal abrasion, we propose that ‘large levels of decomposition and transport’ did not occur. It is therefore most likely parautochthonous.

#### 5.1.4. Paratype (QM F44319): partial mandible and maxilla

The preserved elements are associated but disarticulated, and excluding the mandible, do not truncate the edge of the concretion. As the tips of the teeth from the left maxilla are in occlusion with the left mandible (figure 5*e*), we propose that the specimen was originally preserved with both the left maxilla and mandible intact, and post-fossilization weathering has resulted in the loss of the upper jaw and other cranial material. The absence of the remainder of the skeleton is more likely due to pre-burial disarticulation and transportation of elements during bloat and float, and less likely to be a result of post-burial weathering or collection bias. The assigned Dodson [20] decomposition classes of H implies a taphonomic history of, ‘early stage decomposition, carcass floating in channel before burial’, and J implies, ‘large levels of decomposition and transport’ had occurred. However, considering it is part of Voorhies Group III and the Lag Group, it most likely was not transported far from its original location of disarticulation from the remainder of the skeleton. The lack of abrasion and minimal weathering of preserved elements also aligns with this interpretation. We therefore classify this specimen as parautochthonous, and had reached the active or advanced decay stage prior to burial.

#### 5.1.5. Provisionally referred specimen QM F58795: caudal vertebrae segment, osteoderms, left and right pedes

The specimen comprises an articulated series of caudal vertebrae as well as articulated hindlimb segments. The preserved skeletal elements are articulated but truncate the edge of the concretion block. There are no signs of macroscavenging (such as tooth drag marks or spiral fractures). It is possible that the majority of the skeleton was articulated at the time of burial, and the cranial elements, forelimbs, proximal portion of the trunk and distal portion of the tail were either destroyed due to post-diagenetic weathering or not found at the field site. It is equally possible that microscavenging around the cloaca (as observed in *C. porosus* carcasses [42]) localized weakening of the connective tissues around the pelvic girdle and allowed the hindlimbs and proximal tail portion to detach as one contiguous skeletal segment. As we propose that the absence of skeletal elements truncating concretion edges does not represent true absence, this specimen more closely aligns with the Dodson [20] decomposition class B (specimen complete or nearly so, some drifting of major elements) with an inferred decay history of, ‘a period of subaqueous exposure in low energy environment before burial. Decomposition has begun on extremities', rather than the initially assigned decomposition class K. A right ungual is preserved slightly out of *in vivo* position to the right phalanx of digit II. We propose that some soft tissue decay must have occurred prior to burial, but not enough soft tissue decay to result in the complete loss of the ungual. This could have occurred during the active decay stage; the high degrees of articulation between and within skeletal units present are more representative of a carcass buried prior to the advanced decay stage. We cannot, however, rule out the possibility that this ungual was in fact dislocated in life. The skeletal elements preserved fall into Voorhies Groups I and II, comprising the more easily transported skeletal elements, but by the definition of Coard *et al*. [33] comprise both Transport and Lag elements. There are no signs of abrasion and minimal weathering of skeletal elements, suggesting that either the carcass was not transported or was protected by soft tissues during transportation. We conclude that the preserved portion of this skeleton was buried during active or advanced decay, while soft tissue could still hold elements in place, and is parautochthonous.

#### 5.1.6. Provisionally referred specimens (QM F58793 and QM F58794): sub-adult or adult, and juvenile

As UQL-ISIS-III is the only site where two individuals of *I. duncani* were found in close proximity, we considered whether the individuals died concurrently or whether they represent a small-scale lag deposit. While the smaller individual, QM F58794, shows a moderate amount of articulation and completeness (28.9% complete, with average articulation scores of 0.8 and P), the larger individual QM F58793 is only represented by partial cranial elements (1% complete, with articulation scores of 0 and D). However, QM F58793 appears to have suffered more post-mortem decay than QM F58794; it most closely resembles the skeletal remains stage, consisting only of elements identified as belonging to Voorhies Group III (lag deposit). The absence of QM F58793 post-cranial elements is likely representative of true absence, as the recovered cranial portion did not truncate the edge of the concretion, nor were its post-crania recovered intermixed with those of QM F58794. We propose that QM F58794 floated with elements drifting from the decaying carcass (although their absence may not be true absence given that articulated skeletal units truncate the block), and then partially disarticulated at the sediment–water interface before burial. The mandible of QM F58794 lies disarticulated and in contact with the dorsal surface of the skull—a similar pattern of mandible displacement has been observed in a bloated and floating *C. porosus* [42]. It is plausible that the disarticulation and movement of skeletal elements occurred as the body sank and contacted the substrate.

The initially assigned Dodson [20] decomposition class of K for QM F58794 may more likely be class F (skull with incomplete articulated skeleton) with an inferred decay history of, ‘continual decomposition during transportation’. QM F58794 also comprises Lag Group elements as well as more easily transported elements (Voorhies I and II), which suggests that it underwent decay in a relatively low energy setting that continued into the advanced decay stage before burial. The assigned Dodson [20] decomposition classes for QM F58793 also align with greater degrees of decay, perhaps into the skeletal stage, with class I inferred as ‘early stage decomposition, carcass floating in channel before burial’, and class J inferred as ‘large levels of decomposition and transport’. The degree of disarticulation and loss of skeletal elements is more representative of two individuals that did not die concurrently but instead represent a small-scale lag deposit or were coincidentally deposited near to one another. Both QM F58793 and QM F58794 are classified as parautochthonous.

### 5.2. Summary of *Isisfordia* fossil taphonomy

The articulated and partially articulated specimens of *I. duncani* from the Winton Formation at Isisford range from autochthonous to parautochthonous (definition after Kidwell *et al*. [8]): while the holotype QM F36211 and paratype QMF34642 are interpreted to be autochthonous, the partially articulated and/or isolated specimens QMF44319, QMF44320, QM F58793, QM F58794 and QM F58795 are classed as parautochthonous. As there are no sites at Isisford where disarticulated skeletal elements from different species or genera are mixed together in a single concretion, we propose that the fossil remains at Isisford do not represent either attrition or catastrophic burial deposits. Only at one site were the remains of two individuals, QM F58793 and QM F58794, found in in the same concretion, which we consider to be either coincidental or a lag deposit. We do not feel these carcasses were trapped in logjams or external plant roots prior to burial. Although there is evidence for large plant material at the fossil locality, none of it is found in close association with these, or with any, *I. duncani* specimens.

The variation in articulation and completeness between *I. duncani* specimens indicates that they do not represent a mass mortality assemblage with multiple individuals dying concurrently. A more parsimonious explanation is that the carcasses had different residence times in the TAZ, and were therefore buried during different stages of decay. Furthermore, some carcasses (including the holotype QM F36211 and paratype QM F34632) appear to have been half buried during decay, resulting in conflicting taphonomic signatures across a single skeleton. Such a scenario is not inconsistent with the current interpretation of the depositional setting, with flood events and repeated deposition of sand and sand-silt packages in a fluvio-deltaic setting. In such an environment, crocodyliforms are likely to have died from time to time and been variously partially or completely buried. We further postulate that specimens showing lower degrees of articulation and completeness coupled with little sign of bone abrasion or weathering represent carcasses that had some soft tissues protecting bone surfaces and therefore did not reach the remains decay stage prior to burial.

A mosaic-cracking pattern (distinct from mosaic weathering of articular surfaces [103]) was observed to varying intensities across all the *I. duncani* specimens. Although the cause of mosaic cracking in vertebrate bone is as yet unknown [103,121,122], Suarez *et al*. [122] suggested it might result from weathering of highly pneumatized or relatively fragile bone. This could explain the presence of mosaic cracking on the skull of QM F58794—a relatively fragile juvenile skull that was also the only specimen to show any sign of bone deformation (figure 5*c*)—however mosaic cracking was observed on more robust bones such as the limb bones of the holotype QM 36211 (figures 3*a* and 5*a*). Others have proposed that mosaic cracking occurs as a result of sub-aerial desiccation and weathering prior to burial [123,124], although mosaic cracks have been observed on marine vertebrate remains and identified as possibly resulting from subaqueous weathering [121]. Although the *I. duncani* specimens were eventually buried in an aquatic setting, we cannot say for certain that their bones were never exposed subaerially prior to burial. Furthermore, some authors suggest that fractures occur in concretionary material due either to dewatering early in concretion growth, production of fatty acids from decaying carcasses, or tensile stresses during later diagenesis [125–130] (note that the majority of these studies focus on shale and mudstone concretions, unlike the sandstones at Isisford). Large fractures are present in the fossil bearing concretions at Isisford, and micro-fractures have been observed in thin section, cutting through calcite cement and framework grains [6] (C Syme 2017, personal observation). The larger fractures transect skeletal elements resulting in transverse and diagonal bone fractures (figures 3 and 4). However, if the cement micro-fractures and bone mosaic cracking are both caused by high pressure during diagenesis, we cannot explain why the micro-fractures penetrate quartz and feldspar grains but do not penetrate lamellar bone. We feel we cannot conclusively state which mechanism is behind the creation of mosaic cracks in the *I. duncani* fossils.

The *I. duncani* carcasses were buried in the calcium saturated waters of deltaic distributary channels or overbank deposits [6,7], which periodically deposited thick sand units. Although the soft tissues continued to decay after burial, skeletal elements did not undergo complete decay and nutrient recycling. This was probably due to the precipitation of calcite cement in pore spaces decreasing both porosity and permeability [6], later forming indurated carbonate concretions. The high degree of articulation coupled with lack of abrasion and minimal bone surface modification of elements also suggests that the *I. duncani* carcasses were not reworked prior to their encapsulation in concretions.

The apparent lack of abraded elements could be the result of collection bias, or could indicate that skeletal elements buried without protective soft tissues did not have calcite cement form around them. It is not clear if decomposing tissues and associated CDIs catalysed the precipitation of calcite cement, as concretions both with and without fossil material recovered from Isisford have similar petrology and geochemistry [6]. However, it is possible that this is simply an artefact of preservation bias, where concretionary material formed without preserving any trace of the soft tissues required to catalyse their formation. Calcium ions in CDIs can result from fatty tissues decaying and forming adipocere [15,131]. This is parsimonious with the results of our taphonomic analysis that the *I. duncani* fossils were buried with at least some soft tissues still intact. Periods of sediment starvation and extended residence times at the transitional area between sulphate reducing and methanogenic zones would have then allowed sufficient time for concretionary calcite cements to grow [6,15,132].

### 5.3. Comparison with other Winton Formation localities versus contemporaneous localities

It is important to consider the taphonomic interpretations of fossils from Isisford in the context of the lower Winton Formation alone, and not inclusive of all Winton Formation fossil assemblages and environs. When describing the overall palaeoenvironment and fossil taxa of the Winton Formation, many authors group the Isisford vertebrate taxa into one assemblage along with fossils found in the upper Winton Formation near the town of Winton and surrounds [1,71,76,81,133–135]. These localities have produced fossils of sauropod and theropod dinosaurs [69–84], freshwater turtles, [89], crocodylomorphs [68,71], lizards [81], lungfish [52,90] and possible non-mammalian cynodonts [136]. However, we now know that the Isisford locality is approximately 6–8 million years older than the vertebrate fossil bearing localities nearer to Winton [53], and the brackish water signature detected in lower Winton Formation concretion cement from Isisford indicates the presence of a distal-fluvio-deltaic regime with an open connection to the Eromanga Sea during the late Early Cretaceous [6]. A recent analysis of stratigraphic correlations across the Eromanga Basin by Tucker *et al*. [7] found that the lower Winton Formation was deposited as part of a transitional marginal marine to distal continental system as the Eromanga Sea regressed westward, while the upper Winton Formation was deposited in a continental fluvial–alluvial system. They therefore suggest that there should be an informal subdivision between the lower (at Isisford) and upper (nearer Winton) portions of the Winton Formation [7]. We propose that the Isisford fossils should be examined in the context of assemblages recovered from contemporaneous units with similar depositional settings and connection to the Eromanga Sea: such as the marine Griman Creek Formation, Bulldog Shale, Oodnadatta Formation, Toolebuc Formation and Allaru Mudstone, and the marine to paralic Mackunda Formation and Bungil Formation. These units have produced fossils of ammonites, belemnites, bivalves, and gastropods [65,137–139], as well as plesiosaurians [88,89,91,140–145], ichthyosaurs [140,146–151], chondrichthyians and osteichthyans [152–160], lungfish [52,87,90,161], marine turtles [85,86,162,163], crocodyliforms [52,164,165], pterosaurs [166–170], avian dinosaurs [171,172], a possible late-surviving dicynodont [173] (but see Agnolin *et al*. [69] for an alternative interpretation) and non-avian dinosaurs [69,70,72,137,174–182].

### 5.4. Distribution of Cretaceous fossil crocodyliforms in Australia

Fossilized remains of crocodyliforms have been recovered from other Lower Cretaceous (Aptian–Albian) and Upper Cretaceous (Cenomanian), relatively higher (approximately 60°–80°) latitude Gondwanan sites in Australia. The middle Eumerella Formation (Otway Group) at Dinosaur Cove (approx. 125–105 Ma, lower Aptian to middle Albian) is older than the Winton Formation at Isisford (approx. 102.2–100.5 Ma, upper Albian), and both the Griman Creek Formation at Lightning Ridge in New South Wales (previously recorded as middle Albian, now considered to be close to the Albian–Cenomanian boundary (P Bell 2017, personal communication)) and the Winton Formation near Winton (93.9–92.5 Ma, upper Cenomanian–lower Turonian) are younger [50,52,53,61,164,181,183,184]. Dinosaur Cove has produced isolated crocodilian skeletal elements (a quadratojugal, vertebrae and osteoderms) [153] (S Salisbury 2004, personal observation). These individuals are estimated to be relatively diminutive in size (adults under 2.5 m total body length) [55,153,154] (S Salisbury 2017, personal observation) compared with extant Australian adult estuarine crocodiles (*Crocodylus porosus*) and freshwater crocodiles (*Crocodylus johnsoni*); *I. duncani* is also relatively diminutive at around 1.1 m in length [2]. Lightning Ridge has also produced isolated and fragmented fossil elements of crocodyliforms (a mandible, dentary, vertebrae, among others), originally referred to as *Crocodilus* (*Botosaurus*) [sic] *selaslophensis* [185] and Crocodilia indet. [164,186]. Isolated crocodylomorph skeletal elements and teeth have additionally been recovered from the upper Winton Formation near Winton [68,71].

Crocodyliform fossils have been used previously as ‘palaeothermometers’ to map past climactic boundaries [187–194]. This method relies on the inference that the distribution of extant crocodilians, between −30 and 35 degrees latitude, is controlled by mean annual temperatures (MAT) of 14.2°C or higher, relatively short cold seasons with monthly means no colder than 5.5°C, and free access to standing bodies of water [187,188]. This has been used to explain the distribution of crocodyliforms at higher latitudes during the Cretaceous [187]. But because historical factors may have also influenced modern crocodilian distribution, other lines of evidence should be used in combination for more accurate palaeoclimate reconstruction, such as the distribution of other extant temperature-dependent taxa as analogues for extinct taxa or geochemical techniques such as stable isotope analysis [187,189].

The presence of autochthonous and parautochthonous adult and juvenile *I. duncani* fossils at Isisford suggests that during the late Albian there existed an environment suitable for these ectothermic crocodyliforms to live and breed. Assuming that modern crocodilian distribution and physiology is appropriately analogous to that of *I. duncani*, this would imply there was a combination not only of temperate conditions (MAT ≥14.2°C with mean winter temperatures ≥5.5°C), but local hydrology and geography allowing for the presence of standing bodies of water (see Markwick [188]). The presence of a deltaic regime at Isisford as proposed by Syme *et al*. [6] and Tucker *et al*. [7] would have provided the required body of standing water that *I. duncani* could use to aid in thermoregulation (as required by crocodyliforms, see Markwick [187,189] and Huchzermeyer [195]). Seasonal cool to cold conditions likely existed at mid to high latitudes during the Aptian–early Albian, with possible freezing between 50–70° S [52,196–199] (although this is debated by some, see Douglas & Williams [200] and Jell & Duncan [201]). Assuming that Isisford was located at 54° south during the late Albian (based on van Hinsbergen *et al*. [202]), local sea temperatures would have been approximately 10–18°C [199,203–207]. The presence of crocodyliforms not only at Isisford but in other mid to high latitude Gondwanan locations during the late Albian, Cenomanian and early Turonian seems to provide further evidence of warming conditions during the mid-Cretaceous.

## 6. Conclusion

The *I. duncani* fossils from Isisford have varying degrees of articulation and completeness, with little sign of weathering and no signs of abrasion. The retention of articulation in less complete specimens does not mirror disarticulation patterns observed in decay experiments using juvenile *C. porosus* carcasses, suggesting that the majority of *I. duncani* specimens did not undergo prolonged periods of floating in water deeper than the dorso-ventral height of the carcass. The lack of bone surface modification in the form of abrasion or other scratches indicates minimal transportation of decaying remains and/or the presence of soft tissues covering bone surfaces during transport. As most disarticulated skeletal elements remained within close proximity to the original skeleton, or isolated elements were deemed to have low transport potential, these specimens seem typical of carcasses buried during active or advanced decay undergoing minimal transport, and are therefore autochthonous or parautochthonous. The *ex situ* nature of the concretions recovered makes the orientation of remains at the time of burial difficult to ascertain, but at least for the holotype QM F36211 we determined the carcass lay on its dorsal surface prior to burial. Previous geological analysis of the Winton Formation sandstone concretions at Isisford indicated these carcasses were buried in an upper to lower deltaic environment, with deposition of thick sand packages during flood events in low-energy distributary channels and overbank deposits. This is parsimonious with the proposed taphonomic history of the *I. duncani* remains found within sandstone concretions. The presence of both juvenile and adult autochthonous and parautochthonous fossils suggest that *I. duncani* inhabited these deltaic waters near the epicontinental Eromanga Sea during the late Early Cretaceous (late Albian), during a period of global warming. Future studies into Gondwanan palaeoenvironments or palaeobiogeographical analyses of crocodyliform habitat preference should refer to *I. duncani* as a brackish water tolerant species.

## Data accessibility

The datasets supporting this article are included as part of the published manuscript as tables 3–5. These are also available on Dryad (https://doi.org/10.5061/dryad.7vg5fd0) [208].
